# 4-{[(*E*)-(4-Chloro­phen­yl)methyl­idene]amino}-3-{2-[4-(2-methyl­prop­yl)phen­yl]eth­yl}-1*H*-1,2,4-triazole-5(4*H*)-thione

**DOI:** 10.1107/S160053680901650X

**Published:** 2009-05-14

**Authors:** Hoong-Kun Fun, Samuel Robinson Jebas, K. V Sujith, Balakrishna Kalluraya

**Affiliations:** a X-ray Crystallography Unit, School of Physics, Universiti Sains Malaysia, 11800 USM, Penang, Malaysia; b Department of Studies in Chemistry, Mangalore University, Mangalagangotri, Mangalore 574 199, India

## Abstract

The asymmetric unit of the title compound, C_21_H_23_ClN_4_S, contains nine crystallographically independent mol­ecules, labelled *A* to *I*. The orientation of the 2-[4-(2-methyl­prop­yl)phen­yl]ethyl unit with respect to the rest of the mol­ecule is significantly different in mol­ecules *E*, *F*, *H* and *I* compared to the other independent mol­ecules. The isobutyl group of mol­ecule *B* is disordered over two orientations, with occupancies of 0.764 (7) and 0.236 (7). The benzene rings of the chloro­phenyl and methyl­propyl­phenyl units form dihedral angles of 21.90 (11) and 71.47 (11)°, respectively, with the triazole ring in mol­ecule *A* [9.15 (11) and 80.37 (11)° in *B*, 7.14 (11) and 84.06 (11)° in *C*, 25.76 (11) and 76.59 (11)° in *D*, 13.68 (11) and 76.82 (10)° in *E*, 8.38 (11) and 69.77 (10)° in *F*, 30.34 (11) and 78.12 (11)° in *G*, 21.20 (11) and 71.58 (10)° in *H*, and 27.65 (11) and 65.23 (11)° in *I*]. In each independent mol­ecule, a C—H⋯S hydrogen bond is observed. The crystal packing is stabilized by N—H⋯S and C—H⋯S hydrogen bonds, and by C—H⋯π inter­actions involving the methyl­propyl­phenyl ring.

## Related literature

For the activity of ibuprofen, see: Amir & Kumar (2007 ▶). For the activity of 1,2,4-triazol-5-one compounds, see: Demirbas *et al.* (2002 ▶, 2004 ▶). For bond-length data, see: Allen *et al.* (1987 ▶). For related structures, see: Fun *et al.* (2008*a* ▶,*b* ▶). For hydrogen-bond motifs, see: Bernstein *et al.* (1995 ▶). For the stability of the temperature controller used in the data collection, see: Cosier & Glazer (1986 ▶).
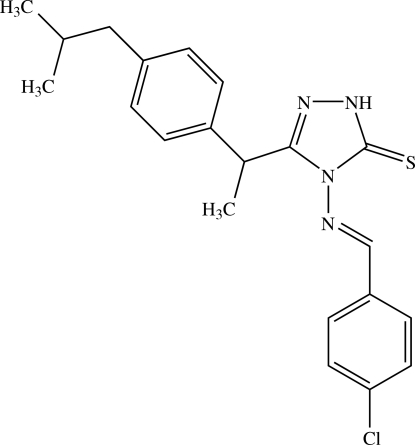


## Experimental

### 

#### Crystal data

C_21_H_23_ClN_4_S
*M* *_r_* = 398.94
Triclinic, 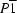

*a* = 13.1781 (2) Å
*b* = 23.7731 (4) Å
*c* = 30.8550 (5) Å
α = 92.861 (1)°
β = 101.520 (1)°
γ = 92.254 (1)°
*V* = 9447.8 (3) Å^3^
*Z* = 18
Mo *K*α radiation
μ = 0.29 mm^−1^
*T* = 100 K
0.55 × 0.34 × 0.13 mm

#### Data collection

Bruker SMART APEXII CCD area-detector diffractometer
Absorption correction: multi-scan (*SADABS*; Bruker, 2005 ▶) *T*_min_ = 0.855, *T*_max_ = 0.962
157626 measured reflections
38430 independent reflections
27090 reflections with *I* > 2σ(*I*)
*R*_int_ = 0.044

#### Refinement

*R*[*F* ^2^ > 2σ(*F* ^2^)] = 0.046
*wR*(*F* ^2^) = 0.139
*S* = 1.01
38430 reflections
2230 parameters
71 restraints
H-atom parameters constrained
Δρ_max_ = 0.84 e Å^−3^
Δρ_min_ = −0.63 e Å^−3^

### 

Data collection: *APEX2* (Bruker, 2005 ▶); cell refinement: *SAINT* (Bruker, 2005 ▶); data reduction: *SAINT*; program(s) used to solve structure: *SHELXTL* (Sheldrick, 2008 ▶); program(s) used to refine structure: *SHELXTL*; molecular graphics: *SHELXTL*; software used to prepare material for publication: *SHELXTL* and *PLATON* (Spek, 2009 ▶).

## Supplementary Material

Crystal structure: contains datablocks global, I. DOI: 10.1107/S160053680901650X/ci2794sup1.cif

Structure factors: contains datablocks I. DOI: 10.1107/S160053680901650X/ci2794Isup2.hkl

Additional supplementary materials:  crystallographic information; 3D view; checkCIF report

## Figures and Tables

**Table 1:** Hydrogen-bond geometry (Å, °)

*D*—H⋯*A*	*D*—H	H⋯*A*	*D*⋯*A*	*D*—H⋯*A*
N1*A*—H1*AB*⋯S1*F*^i^	0.86	2.52	3.373 (2)	170
N1*B*—H1*BB*⋯S1*C*^ii^	0.86	2.47	3.3072 (19)	163
N1*C*—H1*CB*⋯S1*B*^iii^	0.86	2.45	3.285 (2)	165
N1*D*—H1*DB*⋯S1*E*^iv^	0.86	2.48	3.3289 (19)	171
N1*E*—H1*EB*⋯S1*D*^v^	0.86	2.41	3.253 (2)	168
N1*F*—H1*FB*⋯S1*A*^i^	0.86	2.41	3.256 (2)	167
N1*G*—H1*GB*⋯S1*H*^iv^	0.86	2.43	3.287 (2)	174
N1*H*—H1*HB*⋯S1*G*^v^	0.86	2.45	3.3047 (19)	170
N1*I*—H1*IB*⋯S1*I*^vi^	0.86	2.45	3.301 (2)	172
C10*D*—H10*D*⋯S1*C*^iv^	0.98	2.87	3.769 (2)	152
C10*G*—H10*G*⋯S1*F*^iv^	0.98	2.82	3.662 (2)	144
C7*A*—H7*AA*⋯S1*A*	0.93	2.58	3.174 (2)	122
C7*B*—H7*BA*⋯S1*B*	0.93	2.57	3.240 (2)	130
C7*C*—H7*CA*⋯S1*C*	0.93	2.58	3.254 (2)	130
C7*D*—H7*DA*⋯S1*D*	0.93	2.59	3.174 (2)	121
C7*E*—H7*EA*⋯S1*E*	0.93	2.56	3.223 (2)	129
C7*F*—H7*FA*⋯S1*F*	0.93	2.56	3.229 (2)	130
C7*G*—H7*GA*⋯S1*G*	0.93	2.61	3.178 (2)	120
C7*H*—H7*HA*⋯S1*H*	0.93	2.55	3.187 (2)	126
C7*I*—H7*IA*⋯S1*I*	0.93	2.61	3.179 (2)	120
C4*D*—H4*DA*⋯*Cg*1	0.93	2.72	3.588 (2)	156
C4*E*—H4*EA*⋯*Cg*2	0.93	2.84	3.566 (3)	136
C4*G*—H4*GA*⋯*Cg*3	0.93	2.86	3.701 (2)	152
C4*H*—H4*HA*⋯*Cg*4	0.93	2.51	3.338 (2)	148
C4*A*—H4*AA*⋯*Cg*5^vi^	0.93	2.72	3.606 (2)	159
C4*C*—H4*CA*⋯*Cg*6^vii^	0.93	2.96	3.670 (2)	134
C4*F*—H4*FA*⋯*Cg*7^vi^	0.93	2.78	3.524 (3)	137
C4*I*—H4*IA*⋯*Cg*8^viii^	0.93	2.88	3.719 (2)	151

Symmetry codes: (i) 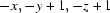; (ii) 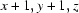; (iii) 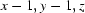; (iv) 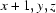; (v) 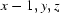; (vi) 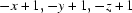; (vii) 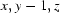; (viii) 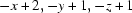. *Cg*1 is the centroid of the N1/C2–C5 ring.

**Fig. 1.**
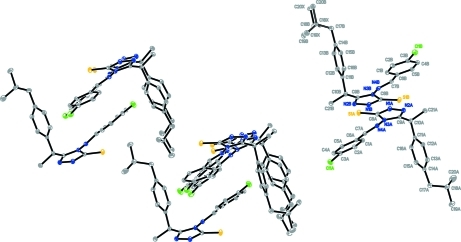
The asymmetric unit of the title compound, showing 30% probability displacement ellipsoids and the atom numbering scheme of molecule A and molecule B. For clarity labelling is not shown for the remaining seven molecules.

**Fig. 2.**
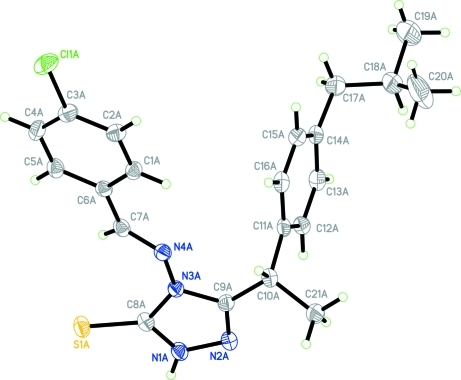
One of the nine independent molecules in the asymmertic unit to show clearly the atomic labelling. The other eight molecules are labelled in a similar manner.

**Fig. 3.**
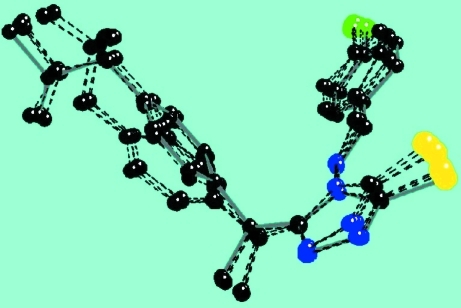
Superimposed fit of molecule A (solid lines) and molecules B to I (dashed lines).

## supplementary crystallographic information

### Comment

1,2,4-Triazoles and their derivatives represent an overwhelming and rapid developing field in modern heterocyclic chemistry. Similarly, ibuprofen belongs to the class of non-steroidal anti-inflammatory drugs (NSAIDs) with antipyretic, anti-inflammatory and analgesic properties (Amir & Kumar, 2007). Our earlier studies involved synthesis of heterocyclic compounds containing in their structures both the ibuprofen and 1,2,4-triazole fragments (Fun *et al.*, 2008*a*,*b*). Schiff base derivatives of 1,2,4-triazol-5-ones are found to possess antitumor activity (Demirbas *et al.*, 2004). Some Schiff base derivatives of acetic acid hydrazides containing 1,2,4-triazol-5-one ring have displayed antitumoral activity against breast cancer, while 2-phenyl ethylidenamino and 2-phenyl ethylamino derivatives of 4-amino-1,2,4-triazol-5-ones have been found to be effective towards non-small cell lung cancer, CNC and breast cancer (Demirbas *et al.*, 2002). In this connection and in continuation of our interest in the synthesis of chemically and biologically important heterocycles, we now report the title compound which is a substituted 1,2,4-triazole Schiff base carrying ibuprofen moiety.

The asymmetric unit of the title compound (Fig. 1) contains nine crystallographically independent molecules (say A to I; their atoms are labelled with suffixes A to I). The orientation of the 1-[4-(2-methylpropyl)phenyl]ethyl unit with respect to the rest of the molecule is different in some of these molecules. This is indicated by the C(H_3_)—C···C—C(H_2_) i.e. the C21—C10···C17—C18 torsion angle of -10.8 (2), 3.2 (2), 7.0 (2), -2.1 (2), 151.60 (18), 156.32 (18), 42.1 (3), 154.01 (18) and 156.10 (18)°, respectively, in molecules A, B, C, D, E, F, G, H and I. This is also clearly shown in a superimposed fit (Fig. 3) of molecule A (solid) and other molecules (dashed lines). Bond lengths (Allen *et al.*, 1987) and angles are normal. The isobutyl group of the molecule B is disordered over two orientations with occupancies of 0.764 (7) and 0.236 (7). The C1–C6 and C11–C16 benzene rings form dihedral angles of 21.90 (11) and 71.47 (11)°, respectively, with the triazole ring in molecule A [9.15 (11)° and 80.37 (11)° in B, 7.14 (11)° and 84.06 (11)° in C, 25.76 (11)° and 76.59 (11)° in D, 13.68 (11)° and 76.82 (10)° in E, 8.38 (11)° and 69.77 (10)° in F, 30.34 (11)° and 78.12 (11)° in G, 21.20 (11)° and 71.58 (10)° in H, and 27.65 (11)° and 65.23 (11)° in molecule I]. In six of the molecules, the C1–C6 and C11–C16 benzene rings are almost perpendicular to each other [dihedral angles are: 87.65 (10)° (A), 89.42 (10)° (B), 89.02 (10)° (C), 89.52 (10) (E), 87.24 (10)° (H) and 87.15 (10)° (I)]. The dihedral angle between the two benzene rings in the other three molecules are 79.74 (10)° (D), 78.13 (10)° (F), and 74.11 (10)° (G). In each independent molecule a C—H···S hydrogen bond is observed (Bernstein *et al.*, 1995).

The crystal packing is stabilized by N—H···S and C—H···S hydrogen bonds, and C—H···π interactions involving the methylpropylphenyl ring.

### Experimental

The title compound, a Schiff base, was obtained by refluxing 4-amino-5-[1-(4-isobutylphenyl)ethyl]-4*H*-1,2,4-triazole-3-thiol (0.01 mol) and 4-chlorobenzaldehyde (0.01 mol) in ethanol (50 ml) by adding 3 drops of concentrated sulfuric acid for 3 h. The solid product obtained was collected by filtration, washed with ethanol and dried. It was then recrystallized using ethanol. Single crystals suitable for X-ray analysis were obtained by slow evaporation of an ethanol–*N*,*N*-dimethylformamide (3:1) solution.

### Refinement

H atoms were positioned geometrically [C—H = 0.93–0.98 Å and N—H = 0.86 Å] and refined using a riding model, with *U*_iso_(H) = 1.2*U*_eq_(C, N) and 1.5*U*_eq_(methyl C). A rotating group model was used for the methyl groups. The methylpropyl unit of molecule B is disordered over two sites with occupancies of 0.764 (7) and 0.236 (7).

### Crystal data

**Table:**

C_21_H_23_ClN_4_S	*Z* = 18
*M**_r_* = 398.94	*F*(000) = 3780
Triclinic, *P*1	*D*_x_ = 1.262 Mg m^−^^3^
Hall symbol: -P 1	Mo *K*α radiation, λ = 0.71073 Å
*a* = 13.1781 (2) Å	Cell parameters from 9483 reflections
*b* = 23.7731 (4) Å	θ = 2.4–30.9°
*c* = 30.8550 (5) Å	µ = 0.29 mm^−^^1^
α = 92.861 (1)°	*T* = 100 K
β = 101.520 (1)°	Block, colourless
γ = 92.254 (1)°	0.55 × 0.34 × 0.13 mm
*V* = 9447.8 (3) Å^3^	

### Data collection

**Table:**

Bruker SMART APEXII CCD area-detector diffractometer	38430 independent reflections
Radiation source: fine-focus sealed tube	27090 reflections with *I* > 2σ(*I*)
graphite	*R*_int_ = 0.044
φ and ω scans	θ_max_ = 26.5°, θ_min_ = 0.7°
Absorption correction: multi-scan (*SADABS*; Bruker, 2005)	*h* = −16→14
*T*_min_ = 0.855, *T*_max_ = 0.962	*k* = −29→29
157626 measured reflections	*l* = −38→38

### Refinement

**Table:**

Refinement on *F*^2^	Primary atom site location: structure-invariant direct methods
Least-squares matrix: full	Secondary atom site location: difference Fourier map
*R*[*F*^2^ > 2σ(*F*^2^)] = 0.046	Hydrogen site location: inferred from neighbouring sites
*wR*(*F*^2^) = 0.139	H-atom parameters constrained
*S* = 1.01	*w* = 1/[σ^2^(*F*_o_^2^) + (0.0694*P*)^2^ + 3.1587*P*] where *P* = (*F*_o_^2^ + 2*F*_c_^2^)/3
38430 reflections	(Δ/σ)_max_ = 0.001
2230 parameters	Δρ_max_ = 0.84 e Å^−^^3^
71 restraints	Δρ_min_ = −0.63 e Å^−^^3^

### Special details

**Table:**

Experimental. The crystal was placed in the cold stream of an Oxford Cyrosystems Cobra open-flow nitrogen cryostat (Cosier & Glazer, 1986) operating at 100.0 (1) K.
Geometry. All e.s.d.'s (except the e.s.d. in the dihedral angle between two l.s. planes) are estimated using the full covariance matrix. The cell e.s.d.'s are taken into account individually in the estimation of e.s.d.'s in distances, angles and torsion angles; correlations between e.s.d.'s in cell parameters are only used when they are defined by crystal symmetry. An approximate (isotropic) treatment of cell e.s.d.'s is used for estimating e.s.d.'s involving l.s. planes.
Refinement. Refinement of *F*^2^ against ALL reflections. The weighted *R*-factor *wR* and goodness of fit *S* are based on *F*^2^, conventional *R*-factors *R* are based on *F*, with *F* set to zero for negative *F*^2^. The threshold expression of *F*^2^ > σ(*F*^2^) is used only for calculating *R*-factors(gt) *etc*. and is not relevant to the choice of reflections for refinement. *R*-factors based on *F*^2^ are statistically about twice as large as those based on *F*, and *R*- factors based on ALL data will be even larger.

### Fractional atomic coordinates and isotropic or equivalent isotropic displacement parameters (Å^2^)

**Table:**

	*x*	*y*	*z*	*U*_iso_*/*U*_eq_	Occ. (<1)
Cl1A	0.85383 (5)	0.82060 (3)	0.703247 (19)	0.03750 (15)	
S1A	0.25870 (5)	0.80270 (2)	0.780325 (18)	0.02729 (13)	
N1A	0.23128 (14)	0.88548 (7)	0.84039 (6)	0.0237 (4)	
H1AB	0.1724	0.8711	0.8434	0.028*	
N2A	0.27375 (14)	0.93578 (7)	0.86153 (6)	0.0246 (4)	
N3A	0.37677 (13)	0.89701 (7)	0.82143 (5)	0.0202 (4)	
N4A	0.46114 (14)	0.89728 (7)	0.80031 (5)	0.0211 (4)	
C1A	0.64370 (17)	0.88998 (9)	0.76293 (7)	0.0231 (5)	
H1AA	0.6300	0.9258	0.7732	0.028*	
C2A	0.72680 (17)	0.88322 (9)	0.74275 (7)	0.0262 (5)	
H2AA	0.7693	0.9141	0.7393	0.031*	
C3A	0.74637 (18)	0.82949 (9)	0.72757 (7)	0.0260 (5)	
C4A	0.68417 (18)	0.78280 (9)	0.73183 (7)	0.0267 (5)	
H4AA	0.6979	0.7472	0.7212	0.032*	
C5A	0.60083 (18)	0.79017 (9)	0.75234 (7)	0.0246 (5)	
H5AA	0.5585	0.7591	0.7556	0.030*	
C6A	0.57929 (16)	0.84360 (8)	0.76826 (6)	0.0202 (4)	
C7A	0.49208 (17)	0.84877 (9)	0.79050 (6)	0.0223 (5)	
H7AA	0.4585	0.8164	0.7977	0.027*	
C8A	0.28930 (17)	0.86102 (9)	0.81480 (6)	0.0217 (5)	
C9A	0.36170 (17)	0.94227 (8)	0.84916 (7)	0.0213 (5)	
C10A	0.43785 (17)	0.99199 (8)	0.86148 (7)	0.0224 (5)	
H10A	0.4649	1.0007	0.8351	0.027*	
C11A	0.52965 (17)	0.97921 (8)	0.89767 (7)	0.0220 (5)	
C12A	0.51540 (18)	0.96624 (9)	0.93983 (7)	0.0249 (5)	
H12A	0.4488	0.9632	0.9456	0.030*	
C13A	0.60008 (18)	0.95797 (9)	0.97309 (7)	0.0278 (5)	
H13A	0.5892	0.9495	1.0010	0.033*	
C14A	0.70079 (18)	0.96204 (9)	0.96593 (7)	0.0288 (5)	
C15A	0.71414 (18)	0.97422 (9)	0.92368 (7)	0.0295 (5)	
H15A	0.7808	0.9770	0.9180	0.035*	
C16A	0.63030 (18)	0.98220 (9)	0.89010 (7)	0.0262 (5)	
H16A	0.6413	0.9897	0.8620	0.031*	
C17A	0.7925 (2)	0.95564 (10)	1.00328 (8)	0.0371 (6)	
H17A	0.7800	0.9219	1.0183	0.045*	
H17B	0.8536	0.9504	0.9908	0.045*	
C18A	0.8142 (2)	1.00507 (11)	1.03694 (8)	0.0423 (7)	
H18A	0.7532	1.0075	1.0505	0.051*	
C19A	0.9063 (2)	0.99401 (14)	1.07425 (10)	0.0664 (10)	
H19A	0.9162	1.0247	1.0964	0.100*	
H19B	0.9678	0.9909	1.0623	0.100*	
H19C	0.8922	0.9595	1.0874	0.100*	
C20A	0.8294 (3)	1.06006 (12)	1.01865 (11)	0.0753 (11)	
H20A	0.7700	1.0668	0.9963	0.113*	
H20B	0.8901	1.0599	1.0058	0.113*	
H20C	0.8382	1.0893	1.0419	0.113*	
C21A	0.38360 (18)	1.04385 (9)	0.87514 (7)	0.0296 (5)	
H21A	0.3294	1.0526	0.8510	0.044*	
H21B	0.4330	1.0753	0.8825	0.044*	
H21C	0.3544	1.0361	0.9005	0.044*	
Cl1B	−0.13778 (5)	0.99741 (3)	0.82181 (2)	0.03966 (16)	
S1B	0.48175 (5)	1.06825 (2)	0.758803 (18)	0.02621 (13)	
N1B	0.51925 (14)	0.98842 (7)	0.69898 (5)	0.0238 (4)	
H1BB	0.5765	1.0052	0.6963	0.029*	
N2B	0.48270 (14)	0.93719 (7)	0.67713 (6)	0.0245 (4)	
N3B	0.37483 (13)	0.97019 (7)	0.71727 (5)	0.0198 (4)	
N4B	0.28658 (13)	0.96490 (7)	0.73551 (5)	0.0208 (4)	
C1B	0.09189 (17)	0.95660 (8)	0.76112 (7)	0.0231 (5)	
H1BA	0.1102	0.9254	0.7455	0.028*	
C2B	0.00098 (18)	0.95409 (9)	0.77678 (7)	0.0262 (5)	
H2BA	−0.0425	0.9217	0.7714	0.031*	
C3B	−0.02473 (18)	1.00067 (10)	0.80065 (7)	0.0266 (5)	
C4B	0.03792 (19)	1.04946 (9)	0.80802 (7)	0.0292 (5)	
H4BA	0.0198	1.0804	0.8240	0.035*	
C5B	0.12753 (18)	1.05204 (9)	0.79153 (7)	0.0261 (5)	
H5BA	0.1691	1.0852	0.7959	0.031*	
C6B	0.15686 (17)	1.00537 (8)	0.76832 (6)	0.0211 (5)	
C7B	0.25204 (17)	1.00978 (9)	0.75097 (6)	0.0220 (5)	
H7BA	0.2871	1.0445	0.7511	0.026*	
C8B	0.45772 (17)	1.00959 (8)	0.72473 (6)	0.0216 (5)	
C9B	0.39551 (17)	0.92678 (8)	0.68903 (6)	0.0210 (5)	
C10B	0.32423 (16)	0.87562 (8)	0.67458 (7)	0.0227 (5)	
H10B	0.3005	0.8623	0.7006	0.027*	
C11B	0.22964 (17)	0.89028 (8)	0.64081 (7)	0.0213 (5)	
C12B	0.24070 (17)	0.91431 (9)	0.60174 (7)	0.0243 (5)	
H12B	0.3067	0.9224	0.5966	0.029*	
C13B	0.15441 (18)	0.92629 (9)	0.57037 (7)	0.0264 (5)	
H13B	0.1636	0.9424	0.5445	0.032*	
C14B	0.05462 (18)	0.91472 (9)	0.57686 (7)	0.0270 (5)	
C15B	0.04343 (18)	0.89046 (9)	0.61604 (7)	0.0300 (5)	
H15B	−0.0226	0.8822	0.6212	0.036*	
C16B	0.13016 (17)	0.87850 (9)	0.64746 (7)	0.0253 (5)	
H16B	0.1212	0.8623	0.6734	0.030*	
C17B	−0.03791 (19)	0.92605 (10)	0.54174 (8)	0.0358 (6)	
H17C	−0.0315	0.9647	0.5336	0.043*	0.764 (7)
H17Q	−0.1000	0.9221	0.5540	0.043*	0.764 (7)
H17R	−0.0145	0.9485	0.5203	0.043*	0.236 (7)
H17S	−0.0835	0.9483	0.5554	0.043*	0.236 (7)
C18B	−0.0503 (3)	0.88723 (13)	0.50063 (10)	0.0308 (10)	0.764 (7)
H18B	0.0062	0.8989	0.4861	0.037*	0.764 (7)
C19B	−0.0382 (4)	0.8256 (2)	0.50700 (19)	0.0389 (12)	0.764 (7)
H19D	0.0303	0.8199	0.5233	0.058*	0.764 (7)
H19E	−0.0489	0.8053	0.4786	0.058*	0.764 (7)
H19F	−0.0884	0.8120	0.5232	0.058*	0.764 (7)
C20B	−0.1495 (4)	0.8974 (2)	0.46773 (15)	0.0476 (12)	0.764 (7)
H20D	−0.1519	0.8750	0.4408	0.071*	0.764 (7)
H20E	−0.1509	0.9366	0.4615	0.071*	0.764 (7)
H20F	−0.2084	0.8872	0.4802	0.071*	0.764 (7)
C18X	−0.0996 (8)	0.8745 (4)	0.5164 (3)	0.017 (3)*	0.236 (7)
H18J	−0.1447	0.8658	0.5372	0.020*	0.236 (7)
C19X	−0.0640 (16)	0.8310 (9)	0.5134 (7)	0.037 (5)*	0.236 (7)
H19G	−0.0630	0.8119	0.5402	0.055*	0.236 (7)
H19H	0.0055	0.8363	0.5087	0.055*	0.236 (7)
H19I	−0.1051	0.8087	0.4888	0.055*	0.236 (7)
C20X	−0.1838 (12)	0.8951 (7)	0.4767 (5)	0.031 (3)*	0.236 (7)
H20G	−0.2331	0.8645	0.4651	0.046*	0.236 (7)
H20H	−0.1501	0.9074	0.4538	0.046*	0.236 (7)
H20I	−0.2191	0.9258	0.4874	0.046*	0.236 (7)
C21B	0.38263 (17)	0.82869 (9)	0.65628 (7)	0.0277 (5)	
H21D	0.4402	0.8195	0.6787	0.041*	
H21E	0.4076	0.8412	0.6310	0.041*	
H21F	0.3368	0.7959	0.6476	0.041*	
Cl1C	0.38051 (5)	0.10472 (3)	0.632486 (19)	0.03874 (15)	
S1C	−0.23891 (4)	0.02693 (2)	0.695205 (17)	0.02521 (13)	
N1C	−0.28244 (14)	0.11008 (7)	0.75088 (5)	0.0232 (4)	
H1CB	−0.3388	0.0928	0.7541	0.028*	
N2C	−0.24996 (14)	0.16287 (7)	0.77044 (6)	0.0244 (4)	
N3C	−0.13860 (13)	0.12876 (7)	0.73237 (5)	0.0199 (4)	
N4C	−0.04987 (13)	0.13417 (7)	0.71452 (5)	0.0201 (4)	
C1C	0.14639 (17)	0.14414 (9)	0.69089 (7)	0.0234 (5)	
H1CA	0.1263	0.1753	0.7059	0.028*	
C2C	0.23754 (17)	0.14752 (9)	0.67526 (7)	0.0261 (5)	
H2CA	0.2789	0.1807	0.6797	0.031*	
C3C	0.26654 (17)	0.10072 (10)	0.65291 (7)	0.0261 (5)	
C4C	0.20609 (18)	0.05100 (9)	0.64595 (7)	0.0279 (5)	
H4CA	0.2261	0.0201	0.6306	0.033*	
C5C	0.11595 (18)	0.04784 (9)	0.66201 (7)	0.0248 (5)	
H5CA	0.0757	0.0142	0.6579	0.030*	
C6C	0.08405 (16)	0.09423 (8)	0.68433 (6)	0.0203 (5)	
C7C	−0.01182 (16)	0.08933 (8)	0.70132 (6)	0.0212 (5)	
H7CA	−0.0445	0.0542	0.7025	0.025*	
C8C	−0.21894 (17)	0.08805 (8)	0.72646 (7)	0.0212 (5)	
C9C	−0.16298 (17)	0.17350 (8)	0.75831 (6)	0.0204 (5)	
C10C	−0.09444 (17)	0.22588 (8)	0.77116 (7)	0.0224 (5)	
H10C	−0.0722	0.2385	0.7446	0.027*	
C11C	0.00160 (17)	0.21418 (8)	0.80516 (7)	0.0226 (5)	
C12C	−0.00735 (18)	0.18729 (9)	0.84341 (7)	0.0254 (5)	
H12C	−0.0726	0.1754	0.8477	0.030*	
C13C	0.07946 (18)	0.17800 (9)	0.87509 (7)	0.0286 (5)	
H13C	0.0716	0.1599	0.9003	0.034*	
C14C	0.17827 (18)	0.19510 (9)	0.87012 (7)	0.0307 (5)	
C15C	0.18699 (19)	0.22212 (10)	0.83171 (7)	0.0336 (6)	
H15C	0.2522	0.2340	0.8275	0.040*	
C16C	0.09996 (18)	0.23145 (10)	0.79982 (7)	0.0298 (5)	
H16C	0.1076	0.2495	0.7745	0.036*	
C17C	0.2725 (2)	0.18702 (11)	0.90596 (8)	0.0385 (6)	
H17D	0.3344	0.1983	0.8954	0.046*	
H17E	0.2759	0.1473	0.9116	0.046*	
C18C	0.2714 (2)	0.22064 (11)	0.94913 (8)	0.0395 (6)	
H18C	0.2154	0.2042	0.9618	0.047*	
C19C	0.2511 (2)	0.28123 (10)	0.94309 (8)	0.0358 (6)	
H19J	0.1824	0.2843	0.9260	0.054*	
H19K	0.3007	0.2976	0.9278	0.054*	
H19L	0.2571	0.3008	0.9715	0.054*	
C20C	0.3740 (2)	0.21397 (12)	0.98213 (9)	0.0560 (8)	
H20J	0.3719	0.2336	1.0099	0.084*	
H20K	0.4306	0.2295	0.9705	0.084*	
H20L	0.3834	0.1747	0.9866	0.084*	
C21C	−0.15519 (17)	0.27256 (9)	0.78850 (7)	0.0273 (5)	
H21G	−0.2145	0.2797	0.7661	0.041*	
H21H	−0.1115	0.3064	0.7957	0.041*	
H21I	−0.1778	0.2609	0.8145	0.041*	
Cl1D	0.37045 (5)	0.28423 (3)	0.74544 (2)	0.04183 (16)	
S1D	0.96227 (5)	0.30506 (2)	0.666660 (19)	0.02873 (14)	
N1D	0.99868 (14)	0.21702 (7)	0.61318 (6)	0.0242 (4)	
H1DB	1.0572	0.2316	0.6098	0.029*	
N2D	0.95998 (14)	0.16449 (7)	0.59513 (6)	0.0239 (4)	
N3D	0.85226 (13)	0.20589 (7)	0.63133 (5)	0.0192 (4)	
N4D	0.76695 (13)	0.20644 (7)	0.65203 (5)	0.0204 (4)	
C1D	0.58706 (17)	0.21390 (9)	0.69013 (7)	0.0234 (5)	
H1DA	0.6055	0.1777	0.6831	0.028*	
C2D	0.50422 (18)	0.22107 (9)	0.71066 (7)	0.0274 (5)	
H2DA	0.4675	0.1899	0.7180	0.033*	
C3D	0.47642 (18)	0.27519 (10)	0.72026 (7)	0.0271 (5)	
C4D	0.53066 (19)	0.32223 (9)	0.71050 (7)	0.0292 (5)	
H4DA	0.5114	0.3583	0.7174	0.035*	
C5D	0.61388 (18)	0.31474 (9)	0.69028 (7)	0.0261 (5)	
H5DA	0.6509	0.3461	0.6835	0.031*	
C6D	0.64357 (17)	0.26058 (8)	0.67978 (6)	0.0216 (5)	
C7D	0.73190 (17)	0.25500 (9)	0.65830 (7)	0.0231 (5)	
H7DA	0.7629	0.2869	0.6491	0.028*	
C8D	0.93738 (17)	0.24321 (8)	0.63626 (7)	0.0221 (5)	
C9D	0.87164 (17)	0.15822 (8)	0.60703 (7)	0.0207 (5)	
C10D	0.79832 (17)	0.10732 (8)	0.59684 (7)	0.0219 (5)	
H10D	0.7750	0.0988	0.6242	0.026*	
C11D	0.70285 (16)	0.11824 (8)	0.56214 (7)	0.0205 (5)	
C12D	0.71159 (17)	0.13153 (9)	0.51955 (7)	0.0245 (5)	
H12D	0.7768	0.1355	0.5126	0.029*	
C13D	0.62404 (18)	0.13894 (9)	0.48753 (7)	0.0259 (5)	
H13D	0.6316	0.1483	0.4594	0.031*	
C14D	0.52500 (17)	0.13275 (8)	0.49657 (7)	0.0237 (5)	
C15D	0.51685 (17)	0.11976 (9)	0.53907 (7)	0.0252 (5)	
H15D	0.4516	0.1155	0.5460	0.030*	
C16D	0.60436 (17)	0.11306 (8)	0.57150 (7)	0.0234 (5)	
H16D	0.5969	0.1050	0.5999	0.028*	
C17D	0.42990 (18)	0.13953 (10)	0.46129 (7)	0.0306 (5)	
H17F	0.4330	0.1774	0.4511	0.037*	
H17G	0.3693	0.1360	0.4746	0.037*	
C18D	0.4155 (2)	0.09812 (10)	0.42165 (8)	0.0373 (6)	
H18D	0.4742	0.1051	0.4072	0.045*	
C19D	0.3172 (2)	0.10925 (12)	0.38805 (9)	0.0489 (7)	
H19M	0.3188	0.1481	0.3809	0.073*	
H19N	0.2575	0.1011	0.4005	0.073*	
H19O	0.3137	0.0856	0.3616	0.073*	
C20D	0.4168 (3)	0.03784 (11)	0.43289 (10)	0.0654 (10)	
H20M	0.4824	0.0310	0.4514	0.098*	
H20N	0.4067	0.0141	0.4061	0.098*	
H20O	0.3621	0.0297	0.4484	0.098*	
C21D	0.85355 (18)	0.05629 (9)	0.58289 (7)	0.0284 (5)	
H21J	0.9129	0.0502	0.6056	0.043*	
H21K	0.8756	0.0630	0.5557	0.043*	
H21L	0.8069	0.0235	0.5786	0.043*	
Cl1E	0.83044 (5)	0.32101 (4)	0.52096 (2)	0.0523 (2)	
S1E	0.23746 (4)	0.26006 (2)	0.605360 (18)	0.02509 (13)	
N1E	0.18327 (14)	0.35298 (7)	0.64801 (6)	0.0245 (4)	
H1EB	0.1299	0.3358	0.6543	0.029*	
N2E	0.20849 (14)	0.40927 (7)	0.65912 (6)	0.0243 (4)	
N3E	0.32237 (13)	0.36989 (7)	0.62506 (5)	0.0191 (4)	
N4E	0.40700 (13)	0.37266 (7)	0.60432 (5)	0.0204 (4)	
C1E	0.59372 (17)	0.37713 (9)	0.57074 (7)	0.0254 (5)	
H1EA	0.5685	0.4117	0.5778	0.031*	
C2E	0.68195 (18)	0.37577 (10)	0.55298 (7)	0.0308 (5)	
H2EA	0.7166	0.4090	0.5482	0.037*	
C3E	0.71792 (18)	0.32367 (11)	0.54239 (7)	0.0316 (6)	
C4E	0.66797 (19)	0.27400 (10)	0.54895 (7)	0.0340 (6)	
H4EA	0.6929	0.2396	0.5413	0.041*	
C5E	0.58016 (18)	0.27578 (9)	0.56701 (7)	0.0281 (5)	
H5EA	0.5461	0.2423	0.5717	0.034*	
C6E	0.54213 (16)	0.32738 (9)	0.57820 (6)	0.0206 (5)	
C7E	0.45121 (17)	0.32657 (8)	0.59876 (7)	0.0216 (5)	
H7EA	0.4258	0.2929	0.6077	0.026*	
C8E	0.24898 (17)	0.32730 (8)	0.62652 (7)	0.0214 (5)	
C9E	0.29259 (16)	0.41873 (8)	0.64459 (6)	0.0203 (5)	
C10E	0.35474 (16)	0.47371 (8)	0.64964 (7)	0.0205 (5)	
H10E	0.3820	0.4782	0.6226	0.025*	
C11E	0.44614 (16)	0.47289 (8)	0.68857 (7)	0.0193 (4)	
C12E	0.42888 (17)	0.47057 (8)	0.73171 (7)	0.0239 (5)	
H12E	0.3613	0.4687	0.7364	0.029*	
C13E	0.51114 (18)	0.47104 (8)	0.76738 (7)	0.0249 (5)	
H13E	0.4979	0.4700	0.7958	0.030*	
C14E	0.61355 (17)	0.47297 (8)	0.76179 (7)	0.0222 (5)	
C15E	0.63021 (17)	0.47492 (9)	0.71866 (7)	0.0242 (5)	
H15E	0.6977	0.4763	0.7139	0.029*	
C16E	0.54792 (17)	0.47485 (8)	0.68273 (7)	0.0225 (5)	
H16E	0.5612	0.4761	0.6543	0.027*	
C17E	0.70236 (18)	0.47314 (9)	0.80112 (7)	0.0288 (5)	
H17H	0.6797	0.4890	0.8270	0.035*	
H17I	0.7585	0.4977	0.7958	0.035*	
C18E	0.74432 (17)	0.41484 (9)	0.81149 (7)	0.0267 (5)	
H18E	0.7640	0.3982	0.7848	0.032*	
C19E	0.84109 (19)	0.42163 (11)	0.84840 (8)	0.0375 (6)	
H19P	0.8693	0.3854	0.8539	0.056*	
H19Q	0.8230	0.4375	0.8749	0.056*	
H19R	0.8918	0.4463	0.8396	0.056*	
C20E	0.66371 (19)	0.37535 (9)	0.82456 (7)	0.0304 (5)	
H20P	0.6036	0.3712	0.8010	0.046*	
H20Q	0.6444	0.3907	0.8510	0.046*	
H20R	0.6921	0.3392	0.8300	0.046*	
C21E	0.28509 (17)	0.52254 (9)	0.65482 (7)	0.0265 (5)	
H21M	0.2309	0.5227	0.6289	0.040*	
H21N	0.3256	0.5575	0.6584	0.040*	
H21O	0.2551	0.5180	0.6804	0.040*	
Cl1F	0.61591 (5)	0.22275 (3)	0.07918 (2)	0.04443 (17)	
S1F	0.01418 (5)	0.15690 (2)	0.156284 (18)	0.02538 (13)	
N1F	−0.03279 (14)	0.24510 (7)	0.20618 (6)	0.0230 (4)	
H1FB	−0.0873	0.2276	0.2112	0.028*	
N2F	−0.00403 (14)	0.29996 (7)	0.22143 (6)	0.0237 (4)	
N3F	0.10801 (13)	0.26323 (7)	0.18493 (5)	0.0191 (4)	
N4F	0.19458 (13)	0.26727 (7)	0.16541 (5)	0.0203 (4)	
C1F	0.38710 (17)	0.27321 (9)	0.13664 (7)	0.0229 (5)	
H1FA	0.3670	0.3067	0.1486	0.028*	
C2F	0.47581 (17)	0.27338 (9)	0.11922 (7)	0.0262 (5)	
H2FA	0.5157	0.3066	0.1195	0.031*	
C3F	0.50449 (18)	0.22315 (10)	0.10123 (7)	0.0274 (5)	
C4F	0.44603 (18)	0.17331 (10)	0.10040 (7)	0.0290 (5)	
H4FA	0.4656	0.1401	0.0879	0.035*	
C5F	0.35829 (17)	0.17378 (9)	0.11835 (7)	0.0252 (5)	
H5FA	0.3192	0.1403	0.1183	0.030*	
C6F	0.32706 (16)	0.22344 (8)	0.13659 (6)	0.0204 (5)	
C7F	0.23444 (16)	0.22115 (8)	0.15585 (6)	0.0209 (5)	
H7FA	0.2048	0.1866	0.1611	0.025*	
C8F	0.03097 (16)	0.22131 (8)	0.18287 (6)	0.0207 (5)	
C9F	0.08118 (17)	0.31028 (8)	0.20781 (6)	0.0208 (5)	
C10F	0.14413 (16)	0.36518 (8)	0.21492 (7)	0.0210 (5)	
H10F	0.1732	0.3707	0.1885	0.025*	
C11F	0.23369 (16)	0.36485 (8)	0.25454 (7)	0.0197 (4)	
C12F	0.21410 (17)	0.36079 (9)	0.29701 (7)	0.0235 (5)	
H12F	0.1461	0.3562	0.3009	0.028*	
C13F	0.29481 (17)	0.36354 (9)	0.33353 (7)	0.0243 (5)	
H13F	0.2799	0.3610	0.3616	0.029*	
C14F	0.39763 (17)	0.36997 (8)	0.32934 (7)	0.0223 (5)	
C15F	0.41655 (17)	0.37352 (9)	0.28657 (7)	0.0238 (5)	
H15F	0.4846	0.3777	0.2827	0.029*	
C16F	0.33629 (17)	0.37100 (8)	0.24988 (7)	0.0225 (5)	
H16F	0.3511	0.3734	0.2218	0.027*	
C17F	0.48446 (18)	0.37296 (9)	0.36946 (7)	0.0286 (5)	
H17J	0.4576	0.3860	0.3951	0.034*	
H17K	0.5370	0.4007	0.3653	0.034*	
C18F	0.53579 (18)	0.31678 (10)	0.37932 (7)	0.0293 (5)	
H18F	0.5596	0.3030	0.3528	0.035*	
C19F	0.6300 (2)	0.32591 (12)	0.41714 (8)	0.0424 (7)	
H19S	0.6626	0.2908	0.4225	0.064*	
H19T	0.6083	0.3398	0.4434	0.064*	
H19U	0.6784	0.3529	0.4093	0.064*	
C20F	0.46005 (19)	0.27271 (10)	0.39009 (8)	0.0334 (6)	
H20S	0.4026	0.2664	0.3655	0.050*	
H20T	0.4352	0.2856	0.4159	0.050*	
H20U	0.4942	0.2381	0.3957	0.050*	
C21F	0.07324 (17)	0.41368 (9)	0.22009 (7)	0.0260 (5)	
H21P	0.0197	0.4140	0.1940	0.039*	
H21Q	0.1133	0.4489	0.2242	0.039*	
H21R	0.0424	0.4085	0.2454	0.039*	
Cl1G	0.58511 (5)	0.40958 (3)	0.18862 (2)	0.04281 (16)	
S1G	1.16825 (4)	0.42348 (2)	0.104365 (18)	0.02504 (13)	
N1G	1.21206 (14)	0.32959 (7)	0.05923 (6)	0.0233 (4)	
H1GB	1.2698	0.3442	0.0550	0.028*	
N2G	1.17793 (14)	0.27500 (7)	0.04536 (6)	0.0231 (4)	
N3G	1.06574 (13)	0.31906 (7)	0.07773 (5)	0.0185 (4)	
N4G	0.98101 (13)	0.32122 (7)	0.09861 (5)	0.0203 (4)	
C1G	0.80628 (17)	0.33359 (9)	0.14017 (7)	0.0240 (5)	
H1GA	0.8292	0.2973	0.1379	0.029*	
C2G	0.72490 (18)	0.34339 (9)	0.16089 (7)	0.0263 (5)	
H2GA	0.6936	0.3141	0.1731	0.032*	
C3G	0.68998 (18)	0.39769 (9)	0.16335 (7)	0.0272 (5)	
C4G	0.73655 (18)	0.44226 (9)	0.14642 (7)	0.0287 (5)	
H4GA	0.7129	0.4784	0.1487	0.034*	
C5G	0.81881 (18)	0.43217 (9)	0.12609 (7)	0.0253 (5)	
H5GA	0.8508	0.4619	0.1146	0.030*	
C6G	0.85492 (16)	0.37790 (9)	0.12243 (6)	0.0214 (5)	
C7G	0.94166 (16)	0.36968 (9)	0.10018 (7)	0.0221 (5)	
H7GA	0.9685	0.3997	0.0871	0.027*	
C8G	1.14795 (16)	0.35804 (9)	0.07979 (6)	0.0209 (5)	
C9G	1.08977 (17)	0.26937 (8)	0.05733 (6)	0.0202 (5)	
C10G	1.01953 (16)	0.21699 (8)	0.04971 (7)	0.0200 (4)	
H10G	0.9909	0.2124	0.0764	0.024*	
C11G	0.92961 (16)	0.22226 (8)	0.01104 (7)	0.0202 (5)	
C12G	0.94703 (18)	0.22974 (9)	−0.03141 (7)	0.0241 (5)	
H12G	1.0145	0.2316	−0.0362	0.029*	
C13G	0.86454 (18)	0.23437 (9)	−0.06671 (7)	0.0279 (5)	
H13G	0.8778	0.2394	−0.0948	0.033*	
C14G	0.76256 (18)	0.23168 (9)	−0.06087 (7)	0.0270 (5)	
C15G	0.74543 (18)	0.22366 (9)	−0.01858 (8)	0.0284 (5)	
H15G	0.6778	0.2213	−0.0139	0.034*	
C16G	0.82765 (17)	0.21918 (8)	0.01690 (7)	0.0242 (5)	
H16G	0.8143	0.2141	0.0450	0.029*	
C17G	0.67432 (19)	0.23786 (10)	−0.09968 (8)	0.0392 (6)	
H17L	0.6276	0.2644	−0.0904	0.047*	
H17M	0.7027	0.2546	−0.1230	0.047*	
C18G	0.6130 (3)	0.18650 (13)	−0.11869 (11)	0.0737 (11)	
H18G	0.5727	0.1778	−0.0962	0.088*	
C19G	0.5293 (2)	0.19908 (13)	−0.15886 (9)	0.0615 (9)	
H19V	0.4831	0.1664	−0.1675	0.092*	
H19W	0.5616	0.2084	−0.1830	0.092*	
H19X	0.4910	0.2303	−0.1511	0.092*	
C20G	0.6627 (2)	0.13471 (10)	−0.12500 (9)	0.0512 (8)	
H20V	0.7050	0.1255	−0.0974	0.077*	
H20W	0.7054	0.1392	−0.1466	0.077*	
H20X	0.6109	0.1049	−0.1353	0.077*	
C21G	1.08169 (17)	0.16519 (9)	0.04283 (7)	0.0270 (5)	
H21S	1.1369	0.1627	0.0681	0.041*	
H21T	1.1102	0.1687	0.0167	0.041*	
H21U	1.0368	0.1318	0.0394	0.041*	
Cl1H	1.02420 (5)	0.40189 (3)	−0.045821 (19)	0.03688 (15)	
S1H	0.43759 (5)	0.37605 (2)	0.041519 (18)	0.02556 (13)	
N1H	0.38960 (14)	0.47216 (7)	0.08227 (6)	0.0239 (4)	
H1HB	0.3366	0.4564	0.0900	0.029*	
N2H	0.41720 (14)	0.52880 (7)	0.09088 (6)	0.0239 (4)	
N3H	0.52718 (13)	0.48497 (7)	0.05740 (5)	0.0196 (4)	
N4H	0.61012 (13)	0.48358 (7)	0.03534 (5)	0.0205 (4)	
C1H	0.79151 (17)	0.47527 (9)	−0.00335 (7)	0.0235 (5)	
H1HA	0.7669	0.5113	−0.0015	0.028*	
C2H	0.87734 (17)	0.46685 (9)	−0.02172 (7)	0.0257 (5)	
H2HA	0.9107	0.4969	−0.0323	0.031*	
C3H	0.91316 (17)	0.41282 (9)	−0.02425 (7)	0.0244 (5)	
C4H	0.86412 (17)	0.36714 (9)	−0.00976 (7)	0.0261 (5)	
H4HA	0.8883	0.3311	−0.0123	0.031*	
C5H	0.77832 (17)	0.37606 (9)	0.00866 (7)	0.0243 (5)	
H5HA	0.7447	0.3457	0.0187	0.029*	
C6H	0.74128 (16)	0.43015 (8)	0.01248 (6)	0.0199 (4)	
C7H	0.65312 (16)	0.43635 (9)	0.03395 (6)	0.0206 (5)	
H7HA	0.6278	0.4056	0.0468	0.025*	
C8H	0.45228 (16)	0.44400 (8)	0.06095 (6)	0.0209 (5)	
C9H	0.50066 (16)	0.53573 (8)	0.07510 (6)	0.0197 (4)	
C10H	0.56260 (16)	0.59010 (8)	0.07582 (7)	0.0211 (5)	
H10H	0.5889	0.5904	0.0483	0.025*	
C11H	0.65567 (16)	0.59353 (8)	0.11423 (7)	0.0194 (4)	
C12H	0.64300 (17)	0.60276 (9)	0.15775 (7)	0.0241 (5)	
H12H	0.5767	0.6059	0.1635	0.029*	
C13H	0.72808 (17)	0.60732 (8)	0.19258 (7)	0.0251 (5)	
H13H	0.7178	0.6133	0.2214	0.030*	
C14H	0.82834 (17)	0.60317 (8)	0.18542 (7)	0.0218 (5)	
C15H	0.84039 (17)	0.59375 (9)	0.14183 (7)	0.0236 (5)	
H15H	0.9067	0.5907	0.1361	0.028*	
C16H	0.75610 (16)	0.58881 (8)	0.10692 (7)	0.0220 (5)	
H16H	0.7665	0.5823	0.0782	0.026*	
C17H	0.92053 (17)	0.60769 (9)	0.22347 (7)	0.0268 (5)	
H17N	0.9006	0.6264	0.2489	0.032*	
H17O	0.9750	0.6311	0.2154	0.032*	
C18H	0.96387 (17)	0.55080 (9)	0.23681 (7)	0.0255 (5)	
H18H	0.9832	0.5320	0.2109	0.031*	
C19H	1.06095 (19)	0.55962 (10)	0.27334 (7)	0.0351 (6)	
H19Y	1.1121	0.5824	0.2631	0.053*	
H19Z	1.0882	0.5237	0.2809	0.053*	
H191	1.0436	0.5782	0.2990	0.053*	
C20H	0.88276 (19)	0.51299 (9)	0.25169 (8)	0.0319 (6)	
H20Y	0.9106	0.4770	0.2583	0.048*	
H20Z	0.8222	0.5080	0.2284	0.048*	
H21V	0.8644	0.5302	0.2777	0.048*	
C21H	0.49287 (17)	0.64007 (9)	0.07671 (7)	0.0266 (5)	
H21W	0.4361	0.6360	0.0517	0.040*	
H21X	0.5324	0.6745	0.0755	0.040*	
H21Y	0.4664	0.6410	0.1035	0.040*	
Cl1I	1.21296 (5)	0.48789 (3)	0.37854 (2)	0.04423 (17)	
S1I	0.63155 (5)	0.47533 (2)	0.466598 (18)	0.02747 (13)	
N1I	0.59003 (14)	0.57092 (7)	0.51038 (6)	0.0244 (4)	
H1IB	0.5357	0.5559	0.5175	0.029*	
N2I	0.62130 (14)	0.62674 (7)	0.52048 (6)	0.0247 (4)	
N3I	0.72941 (13)	0.58167 (7)	0.48649 (5)	0.0201 (4)	
N4I	0.81187 (13)	0.57847 (7)	0.46423 (5)	0.0213 (4)	
C1I	0.98724 (17)	0.56479 (9)	0.42320 (7)	0.0248 (5)	
H1IA	0.9624	0.6009	0.4239	0.030*	
C2I	1.06996 (18)	0.55470 (9)	0.40310 (7)	0.0268 (5)	
H2IA	1.1006	0.5835	0.3901	0.032*	
C3I	1.10663 (18)	0.50063 (10)	0.40270 (7)	0.0282 (5)	
C4I	1.06109 (18)	0.45684 (9)	0.42097 (7)	0.0284 (5)	
H4IA	1.0858	0.4208	0.4199	0.034*	
C5I	0.97828 (17)	0.46743 (9)	0.44087 (7)	0.0254 (5)	
H5IA	0.9473	0.4382	0.4533	0.030*	
C6I	0.94023 (16)	0.52160 (8)	0.44255 (6)	0.0207 (5)	
C7I	0.85423 (16)	0.53067 (8)	0.46493 (7)	0.0214 (5)	
H7IA	0.8298	0.5016	0.4798	0.026*	
C8I	0.65090 (17)	0.54208 (9)	0.48856 (7)	0.0226 (5)	
C9I	0.70565 (17)	0.63272 (8)	0.50505 (7)	0.0216 (5)	
C10I	0.76918 (16)	0.68664 (8)	0.50607 (7)	0.0212 (5)	
H10I	0.7968	0.6864	0.4788	0.025*	
C11I	0.86090 (16)	0.69193 (8)	0.54503 (7)	0.0193 (4)	
C12I	0.84706 (18)	0.70529 (9)	0.58789 (7)	0.0253 (5)	
H12I	0.7804	0.7090	0.5931	0.030*	
C13I	0.93140 (18)	0.71319 (9)	0.62292 (7)	0.0268 (5)	
H13I	0.9202	0.7220	0.6512	0.032*	
C14I	1.03210 (17)	0.70825 (8)	0.61671 (7)	0.0231 (5)	
C15I	1.04555 (18)	0.69423 (9)	0.57389 (7)	0.0261 (5)	
H15I	1.1122	0.6904	0.5688	0.031*	
C16I	0.96180 (17)	0.68593 (9)	0.53876 (7)	0.0239 (5)	
H16I	0.9730	0.6762	0.5106	0.029*	
C17I	1.12368 (18)	0.71675 (9)	0.65488 (7)	0.0282 (5)	
H17T	1.1016	0.7359	0.6797	0.034*	
H17U	1.1760	0.7411	0.6462	0.034*	
C18I	1.17252 (18)	0.66182 (9)	0.67021 (7)	0.0273 (5)	
H18I	1.1952	0.6430	0.6451	0.033*	
C19I	1.2675 (2)	0.67446 (11)	0.70738 (8)	0.0379 (6)	
H192	1.2997	0.6399	0.7154	0.057*	
H193	1.2465	0.6920	0.7327	0.057*	
H194	1.3160	0.6994	0.6974	0.057*	
C20I	1.0944 (2)	0.62250 (9)	0.68511 (8)	0.0346 (6)	
H21Z	1.1260	0.5879	0.6934	0.052*	
H22A	1.0357	0.6147	0.6613	0.052*	
H22B	1.0721	0.6400	0.7101	0.052*	
C21I	0.69959 (17)	0.73684 (9)	0.50584 (7)	0.0252 (5)	
H22C	0.6443	0.7327	0.4802	0.038*	
H22D	0.7398	0.7712	0.5050	0.038*	
H22E	0.6711	0.7380	0.5322	0.038*	

### Atomic displacement parameters (Å^2^)

**Table:**

	*U*^11^	*U*^22^	*U*^33^	*U*^12^	*U*^13^	*U*^23^
Cl1A	0.0357 (4)	0.0464 (4)	0.0350 (3)	0.0158 (3)	0.0155 (3)	0.0025 (3)
S1A	0.0278 (3)	0.0259 (3)	0.0285 (3)	−0.0075 (2)	0.0104 (2)	−0.0070 (2)
N1A	0.0207 (11)	0.0243 (9)	0.0258 (9)	−0.0042 (8)	0.0058 (8)	−0.0031 (7)
N2A	0.0250 (11)	0.0230 (9)	0.0242 (9)	−0.0010 (8)	0.0029 (8)	−0.0034 (7)
N3A	0.0186 (10)	0.0210 (9)	0.0207 (9)	−0.0006 (7)	0.0044 (7)	−0.0012 (7)
N4A	0.0197 (10)	0.0246 (9)	0.0191 (9)	0.0000 (8)	0.0043 (7)	0.0000 (7)
C1A	0.0269 (13)	0.0203 (11)	0.0214 (11)	0.0039 (9)	0.0027 (9)	0.0008 (8)
C2A	0.0243 (13)	0.0263 (12)	0.0281 (12)	0.0038 (10)	0.0047 (10)	0.0040 (9)
C3A	0.0274 (14)	0.0336 (12)	0.0178 (10)	0.0086 (10)	0.0053 (9)	0.0022 (9)
C4A	0.0360 (15)	0.0237 (11)	0.0197 (11)	0.0100 (10)	0.0024 (10)	−0.0006 (9)
C5A	0.0299 (14)	0.0221 (11)	0.0200 (10)	0.0013 (9)	0.0003 (9)	0.0014 (8)
C6A	0.0196 (12)	0.0248 (11)	0.0153 (10)	0.0022 (9)	0.0011 (8)	0.0008 (8)
C7A	0.0244 (13)	0.0206 (11)	0.0197 (10)	−0.0005 (9)	−0.0010 (9)	0.0021 (8)
C8A	0.0236 (13)	0.0232 (11)	0.0181 (10)	−0.0027 (9)	0.0047 (9)	0.0014 (8)
C9A	0.0226 (13)	0.0200 (10)	0.0205 (10)	0.0029 (9)	0.0022 (9)	0.0008 (8)
C10A	0.0252 (13)	0.0211 (11)	0.0204 (10)	−0.0015 (9)	0.0047 (9)	−0.0012 (8)
C11A	0.0251 (13)	0.0153 (10)	0.0239 (11)	−0.0014 (9)	0.0027 (9)	−0.0038 (8)
C12A	0.0230 (13)	0.0238 (11)	0.0270 (11)	−0.0013 (9)	0.0048 (10)	−0.0033 (9)
C13A	0.0348 (15)	0.0229 (11)	0.0248 (11)	0.0006 (10)	0.0046 (10)	−0.0016 (9)
C14A	0.0287 (14)	0.0196 (11)	0.0339 (13)	0.0033 (10)	−0.0019 (10)	−0.0075 (9)
C15A	0.0239 (14)	0.0283 (12)	0.0351 (13)	0.0024 (10)	0.0053 (10)	−0.0067 (10)
C16A	0.0318 (14)	0.0219 (11)	0.0249 (11)	−0.0018 (10)	0.0078 (10)	−0.0041 (9)
C17A	0.0360 (16)	0.0351 (13)	0.0356 (13)	0.0107 (11)	−0.0045 (11)	−0.0034 (11)
C18A	0.0392 (17)	0.0420 (15)	0.0381 (14)	0.0069 (12)	−0.0090 (12)	−0.0080 (12)
C19A	0.061 (2)	0.072 (2)	0.0506 (18)	0.0190 (17)	−0.0242 (16)	−0.0159 (16)
C20A	0.087 (3)	0.0431 (18)	0.076 (2)	−0.0200 (17)	−0.027 (2)	−0.0015 (16)
C21A	0.0308 (14)	0.0229 (11)	0.0323 (12)	0.0032 (10)	0.0000 (10)	−0.0003 (9)
Cl1B	0.0323 (4)	0.0573 (4)	0.0332 (3)	0.0098 (3)	0.0144 (3)	0.0024 (3)
S1B	0.0276 (3)	0.0255 (3)	0.0246 (3)	−0.0095 (2)	0.0070 (2)	−0.0059 (2)
N1B	0.0206 (11)	0.0253 (9)	0.0243 (9)	−0.0070 (8)	0.0048 (8)	−0.0035 (7)
N2B	0.0242 (11)	0.0235 (9)	0.0234 (9)	−0.0033 (8)	0.0010 (8)	−0.0036 (7)
N3B	0.0190 (10)	0.0217 (9)	0.0177 (8)	−0.0033 (7)	0.0022 (7)	0.0000 (7)
N4B	0.0184 (10)	0.0257 (9)	0.0177 (9)	−0.0026 (8)	0.0023 (7)	0.0021 (7)
C1B	0.0253 (13)	0.0192 (10)	0.0244 (11)	0.0019 (9)	0.0033 (9)	0.0011 (8)
C2B	0.0252 (13)	0.0263 (12)	0.0274 (11)	0.0019 (10)	0.0050 (10)	0.0066 (9)
C3B	0.0247 (13)	0.0355 (13)	0.0205 (11)	0.0075 (10)	0.0049 (9)	0.0063 (9)
C4B	0.0350 (15)	0.0292 (12)	0.0220 (11)	0.0094 (10)	0.0020 (10)	−0.0030 (9)
C5B	0.0289 (14)	0.0219 (11)	0.0250 (11)	0.0006 (9)	−0.0001 (10)	0.0005 (9)
C6B	0.0221 (12)	0.0218 (11)	0.0181 (10)	0.0008 (9)	0.0006 (9)	0.0024 (8)
C7B	0.0236 (13)	0.0200 (10)	0.0201 (10)	−0.0020 (9)	−0.0003 (9)	0.0022 (8)
C8B	0.0210 (12)	0.0227 (11)	0.0199 (10)	−0.0041 (9)	0.0016 (9)	0.0036 (8)
C9B	0.0207 (13)	0.0230 (11)	0.0181 (10)	0.0006 (9)	0.0016 (9)	0.0007 (8)
C10B	0.0223 (13)	0.0201 (10)	0.0238 (11)	−0.0025 (9)	0.0015 (9)	−0.0009 (8)
C11B	0.0235 (13)	0.0168 (10)	0.0222 (11)	−0.0003 (9)	0.0025 (9)	−0.0037 (8)
C12B	0.0212 (13)	0.0237 (11)	0.0270 (11)	−0.0032 (9)	0.0040 (9)	−0.0023 (9)
C13B	0.0335 (15)	0.0209 (11)	0.0230 (11)	−0.0019 (10)	0.0024 (10)	0.0000 (9)
C14B	0.0261 (14)	0.0219 (11)	0.0301 (12)	0.0044 (9)	0.0005 (10)	−0.0085 (9)
C15B	0.0229 (13)	0.0342 (13)	0.0319 (12)	0.0001 (10)	0.0054 (10)	−0.0075 (10)
C16B	0.0244 (13)	0.0258 (11)	0.0248 (11)	−0.0040 (9)	0.0052 (10)	−0.0031 (9)
C17B	0.0306 (15)	0.0362 (14)	0.0372 (14)	0.0087 (11)	−0.0009 (11)	−0.0025 (11)
C18B	0.031 (2)	0.0341 (18)	0.0262 (17)	−0.0017 (14)	0.0021 (14)	0.0033 (13)
C19B	0.029 (3)	0.033 (2)	0.049 (3)	−0.003 (2)	0.000 (2)	−0.0151 (18)
C20B	0.038 (3)	0.068 (3)	0.033 (2)	0.017 (2)	−0.005 (2)	−0.0003 (19)
C21B	0.0259 (13)	0.0214 (11)	0.0325 (12)	0.0011 (9)	−0.0010 (10)	−0.0019 (9)
Cl1C	0.0294 (4)	0.0600 (4)	0.0309 (3)	0.0093 (3)	0.0140 (3)	0.0042 (3)
S1C	0.0275 (3)	0.0221 (3)	0.0255 (3)	−0.0078 (2)	0.0071 (2)	−0.0038 (2)
N1C	0.0199 (10)	0.0236 (9)	0.0253 (9)	−0.0060 (8)	0.0048 (8)	−0.0031 (7)
N2C	0.0234 (11)	0.0232 (9)	0.0250 (9)	−0.0023 (8)	0.0028 (8)	−0.0028 (7)
N3C	0.0212 (10)	0.0185 (9)	0.0194 (9)	−0.0032 (7)	0.0037 (7)	0.0000 (7)
N4C	0.0163 (10)	0.0232 (9)	0.0209 (9)	−0.0028 (7)	0.0045 (7)	0.0012 (7)
C1C	0.0250 (13)	0.0192 (10)	0.0259 (11)	0.0027 (9)	0.0048 (9)	−0.0001 (9)
C2C	0.0224 (13)	0.0265 (12)	0.0297 (12)	0.0010 (9)	0.0049 (10)	0.0054 (9)
C3C	0.0232 (13)	0.0375 (13)	0.0189 (10)	0.0081 (10)	0.0046 (9)	0.0070 (9)
C4C	0.0332 (15)	0.0294 (12)	0.0205 (11)	0.0099 (10)	0.0033 (10)	−0.0030 (9)
C5C	0.0285 (14)	0.0224 (11)	0.0212 (11)	0.0038 (9)	−0.0009 (9)	0.0005 (8)
C6C	0.0214 (12)	0.0236 (11)	0.0155 (10)	0.0029 (9)	0.0025 (9)	0.0023 (8)
C7C	0.0226 (13)	0.0193 (10)	0.0194 (10)	−0.0030 (9)	−0.0003 (9)	0.0015 (8)
C8C	0.0210 (12)	0.0220 (11)	0.0199 (10)	−0.0021 (9)	0.0029 (9)	0.0026 (8)
C9C	0.0195 (12)	0.0217 (11)	0.0194 (10)	0.0008 (9)	0.0029 (9)	0.0002 (8)
C10C	0.0240 (13)	0.0202 (10)	0.0228 (11)	−0.0024 (9)	0.0056 (9)	−0.0016 (8)
C11C	0.0232 (13)	0.0194 (10)	0.0243 (11)	−0.0006 (9)	0.0046 (9)	−0.0055 (8)
C12C	0.0250 (13)	0.0219 (11)	0.0274 (11)	−0.0054 (9)	0.0032 (10)	−0.0038 (9)
C13C	0.0344 (15)	0.0207 (11)	0.0277 (12)	−0.0005 (10)	0.0006 (10)	−0.0037 (9)
C14C	0.0280 (14)	0.0306 (12)	0.0304 (12)	0.0066 (10)	0.0011 (10)	−0.0120 (10)
C15C	0.0215 (14)	0.0480 (15)	0.0313 (13)	−0.0022 (11)	0.0086 (10)	−0.0098 (11)
C16C	0.0271 (14)	0.0376 (13)	0.0238 (11)	−0.0019 (10)	0.0054 (10)	−0.0048 (10)
C17C	0.0301 (15)	0.0505 (16)	0.0327 (13)	0.0126 (12)	0.0007 (11)	−0.0052 (11)
C18C	0.0409 (17)	0.0408 (15)	0.0329 (13)	0.0005 (12)	−0.0017 (12)	0.0015 (11)
C19C	0.0377 (16)	0.0370 (14)	0.0304 (13)	−0.0084 (11)	0.0043 (11)	−0.0032 (10)
C20C	0.060 (2)	0.0511 (18)	0.0441 (16)	0.0034 (15)	−0.0195 (15)	0.0040 (13)
C21C	0.0242 (13)	0.0227 (11)	0.0339 (12)	0.0010 (9)	0.0041 (10)	−0.0019 (9)
Cl1D	0.0416 (4)	0.0476 (4)	0.0427 (4)	0.0160 (3)	0.0221 (3)	−0.0010 (3)
S1D	0.0286 (3)	0.0255 (3)	0.0324 (3)	−0.0097 (2)	0.0119 (3)	−0.0102 (2)
N1D	0.0212 (11)	0.0228 (9)	0.0285 (10)	−0.0054 (8)	0.0077 (8)	−0.0048 (7)
N2D	0.0216 (11)	0.0221 (9)	0.0266 (10)	−0.0014 (8)	0.0036 (8)	−0.0038 (7)
N3D	0.0192 (10)	0.0184 (9)	0.0200 (9)	−0.0028 (7)	0.0053 (7)	−0.0017 (7)
N4D	0.0176 (10)	0.0238 (9)	0.0188 (9)	−0.0018 (7)	0.0029 (7)	−0.0018 (7)
C1D	0.0246 (13)	0.0213 (11)	0.0237 (11)	0.0030 (9)	0.0040 (9)	−0.0012 (8)
C2D	0.0289 (14)	0.0255 (12)	0.0283 (12)	0.0019 (10)	0.0067 (10)	0.0020 (9)
C3D	0.0252 (13)	0.0351 (13)	0.0222 (11)	0.0100 (10)	0.0071 (9)	−0.0028 (9)
C4D	0.0372 (15)	0.0242 (12)	0.0253 (11)	0.0115 (10)	0.0034 (10)	−0.0029 (9)
C5D	0.0307 (14)	0.0215 (11)	0.0248 (11)	0.0028 (10)	0.0029 (10)	−0.0019 (9)
C6D	0.0218 (12)	0.0235 (11)	0.0176 (10)	0.0016 (9)	0.0003 (9)	−0.0029 (8)
C7D	0.0234 (13)	0.0208 (11)	0.0222 (11)	−0.0021 (9)	−0.0007 (9)	−0.0018 (8)
C8D	0.0215 (13)	0.0214 (11)	0.0226 (11)	−0.0014 (9)	0.0037 (9)	0.0001 (8)
C9D	0.0211 (13)	0.0188 (10)	0.0209 (10)	0.0002 (9)	0.0017 (9)	−0.0009 (8)
C10D	0.0248 (13)	0.0199 (10)	0.0203 (10)	−0.0013 (9)	0.0039 (9)	−0.0009 (8)
C11D	0.0208 (12)	0.0165 (10)	0.0237 (11)	−0.0018 (9)	0.0051 (9)	−0.0043 (8)
C12D	0.0212 (13)	0.0281 (12)	0.0243 (11)	−0.0031 (9)	0.0062 (9)	−0.0017 (9)
C13D	0.0299 (14)	0.0274 (12)	0.0195 (11)	−0.0018 (10)	0.0038 (10)	0.0012 (9)
C14D	0.0234 (13)	0.0198 (11)	0.0262 (11)	0.0015 (9)	0.0021 (9)	−0.0022 (9)
C15D	0.0196 (13)	0.0255 (11)	0.0312 (12)	−0.0014 (9)	0.0086 (10)	−0.0030 (9)
C16D	0.0267 (13)	0.0223 (11)	0.0213 (11)	−0.0023 (9)	0.0058 (9)	−0.0007 (8)
C17D	0.0289 (14)	0.0312 (12)	0.0300 (12)	0.0047 (10)	0.0019 (10)	0.0008 (10)
C18D	0.0345 (15)	0.0368 (14)	0.0350 (13)	0.0036 (11)	−0.0049 (11)	−0.0042 (11)
C19D	0.0443 (18)	0.0543 (17)	0.0391 (15)	0.0077 (14)	−0.0124 (13)	−0.0045 (13)
C20D	0.080 (2)	0.0307 (15)	0.064 (2)	−0.0105 (15)	−0.0362 (17)	0.0045 (13)
C21D	0.0261 (14)	0.0229 (11)	0.0335 (12)	−0.0011 (9)	0.0015 (10)	−0.0037 (9)
Cl1E	0.0261 (4)	0.0951 (6)	0.0372 (4)	0.0076 (4)	0.0134 (3)	−0.0141 (4)
S1E	0.0274 (3)	0.0191 (3)	0.0300 (3)	−0.0041 (2)	0.0109 (2)	−0.0024 (2)
N1E	0.0218 (11)	0.0224 (9)	0.0297 (10)	−0.0046 (8)	0.0083 (8)	−0.0031 (8)
N2E	0.0212 (11)	0.0221 (9)	0.0293 (10)	−0.0024 (8)	0.0067 (8)	−0.0036 (7)
N3E	0.0173 (10)	0.0179 (9)	0.0222 (9)	−0.0011 (7)	0.0045 (7)	0.0008 (7)
N4E	0.0167 (10)	0.0234 (9)	0.0222 (9)	0.0004 (7)	0.0063 (7)	0.0010 (7)
C1E	0.0237 (13)	0.0261 (11)	0.0269 (11)	0.0051 (9)	0.0048 (10)	0.0039 (9)
C2E	0.0265 (14)	0.0397 (14)	0.0273 (12)	0.0024 (11)	0.0064 (10)	0.0069 (10)
C3E	0.0192 (13)	0.0550 (16)	0.0208 (11)	0.0088 (11)	0.0043 (9)	−0.0032 (10)
C4E	0.0294 (15)	0.0393 (14)	0.0315 (13)	0.0139 (11)	0.0031 (11)	−0.0109 (11)
C5E	0.0275 (14)	0.0250 (12)	0.0298 (12)	0.0063 (10)	0.0014 (10)	−0.0035 (9)
C6E	0.0179 (12)	0.0252 (11)	0.0182 (10)	0.0032 (9)	0.0021 (9)	−0.0004 (8)
C7E	0.0227 (13)	0.0187 (10)	0.0228 (11)	0.0006 (9)	0.0028 (9)	0.0016 (8)
C8E	0.0205 (12)	0.0229 (11)	0.0206 (10)	−0.0011 (9)	0.0036 (9)	0.0020 (8)
C9E	0.0187 (12)	0.0210 (10)	0.0209 (10)	0.0023 (9)	0.0035 (9)	−0.0003 (8)
C10E	0.0202 (12)	0.0170 (10)	0.0249 (11)	−0.0006 (9)	0.0066 (9)	−0.0008 (8)
C11E	0.0199 (12)	0.0128 (9)	0.0256 (11)	0.0017 (8)	0.0060 (9)	−0.0017 (8)
C12E	0.0204 (13)	0.0233 (11)	0.0300 (12)	0.0030 (9)	0.0102 (10)	0.0000 (9)
C13E	0.0336 (14)	0.0191 (11)	0.0242 (11)	0.0044 (9)	0.0107 (10)	0.0007 (8)
C14E	0.0225 (13)	0.0157 (10)	0.0279 (11)	−0.0004 (9)	0.0043 (9)	0.0004 (8)
C15E	0.0187 (12)	0.0231 (11)	0.0314 (12)	−0.0028 (9)	0.0076 (10)	0.0011 (9)
C16E	0.0229 (13)	0.0209 (11)	0.0248 (11)	−0.0020 (9)	0.0079 (9)	0.0022 (8)
C17E	0.0293 (14)	0.0259 (12)	0.0289 (12)	−0.0026 (10)	0.0013 (10)	−0.0001 (9)
C18E	0.0260 (13)	0.0329 (12)	0.0221 (11)	0.0055 (10)	0.0071 (9)	0.0002 (9)
C19E	0.0312 (15)	0.0457 (15)	0.0341 (13)	0.0092 (12)	0.0015 (11)	0.0032 (11)
C20E	0.0371 (15)	0.0266 (12)	0.0289 (12)	0.0047 (10)	0.0097 (11)	0.0020 (9)
C21E	0.0230 (13)	0.0223 (11)	0.0343 (12)	0.0033 (9)	0.0050 (10)	0.0015 (9)
Cl1F	0.0291 (4)	0.0704 (5)	0.0380 (3)	0.0105 (3)	0.0165 (3)	−0.0011 (3)
S1F	0.0295 (3)	0.0197 (3)	0.0282 (3)	−0.0033 (2)	0.0103 (2)	−0.0012 (2)
N1F	0.0211 (11)	0.0208 (9)	0.0273 (10)	−0.0040 (8)	0.0071 (8)	−0.0019 (7)
N2F	0.0221 (11)	0.0214 (9)	0.0269 (9)	−0.0015 (8)	0.0044 (8)	−0.0024 (7)
N3F	0.0188 (10)	0.0176 (8)	0.0216 (9)	−0.0004 (7)	0.0060 (7)	0.0008 (7)
N4F	0.0182 (10)	0.0237 (9)	0.0199 (9)	0.0012 (7)	0.0058 (7)	0.0007 (7)
C1F	0.0219 (13)	0.0223 (11)	0.0245 (11)	0.0056 (9)	0.0035 (9)	0.0013 (9)
C2F	0.0238 (13)	0.0293 (12)	0.0262 (11)	0.0015 (10)	0.0050 (10)	0.0059 (9)
C3F	0.0221 (13)	0.0424 (14)	0.0191 (11)	0.0089 (10)	0.0058 (9)	0.0032 (9)
C4F	0.0320 (15)	0.0331 (13)	0.0208 (11)	0.0131 (11)	0.0016 (10)	−0.0041 (9)
C5F	0.0265 (13)	0.0209 (11)	0.0268 (11)	0.0062 (9)	0.0017 (10)	−0.0005 (9)
C6F	0.0192 (12)	0.0223 (11)	0.0194 (10)	0.0033 (9)	0.0027 (9)	0.0024 (8)
C7F	0.0201 (12)	0.0195 (10)	0.0220 (10)	−0.0007 (9)	0.0019 (9)	0.0019 (8)
C8F	0.0199 (12)	0.0215 (11)	0.0204 (10)	0.0008 (9)	0.0028 (9)	0.0052 (8)
C9F	0.0200 (12)	0.0213 (11)	0.0204 (10)	0.0022 (9)	0.0020 (9)	0.0006 (8)
C10F	0.0217 (12)	0.0202 (10)	0.0214 (10)	0.0007 (9)	0.0051 (9)	0.0005 (8)
C11F	0.0204 (12)	0.0136 (10)	0.0255 (11)	0.0009 (8)	0.0060 (9)	−0.0006 (8)
C12F	0.0196 (12)	0.0253 (11)	0.0278 (11)	0.0033 (9)	0.0097 (9)	0.0000 (9)
C13F	0.0287 (14)	0.0236 (11)	0.0229 (11)	0.0033 (9)	0.0106 (10)	0.0010 (9)
C14F	0.0233 (13)	0.0174 (10)	0.0246 (11)	−0.0015 (9)	0.0023 (9)	−0.0008 (8)
C15F	0.0168 (12)	0.0243 (11)	0.0307 (12)	−0.0049 (9)	0.0073 (9)	0.0008 (9)
C16F	0.0266 (13)	0.0196 (10)	0.0225 (11)	−0.0037 (9)	0.0090 (9)	−0.0007 (8)
C17F	0.0263 (14)	0.0286 (12)	0.0289 (12)	−0.0046 (10)	0.0022 (10)	0.0008 (9)
C18F	0.0247 (14)	0.0399 (14)	0.0240 (11)	0.0057 (10)	0.0062 (10)	0.0011 (10)
C19F	0.0321 (16)	0.0569 (17)	0.0358 (14)	0.0064 (13)	−0.0003 (12)	0.0064 (12)
C20F	0.0380 (16)	0.0311 (13)	0.0326 (13)	0.0079 (11)	0.0089 (11)	0.0048 (10)
C21F	0.0235 (13)	0.0214 (11)	0.0330 (12)	0.0048 (9)	0.0048 (10)	0.0018 (9)
Cl1G	0.0428 (4)	0.0482 (4)	0.0446 (4)	0.0182 (3)	0.0234 (3)	0.0004 (3)
S1G	0.0277 (3)	0.0206 (3)	0.0276 (3)	−0.0052 (2)	0.0100 (2)	−0.0041 (2)
N1G	0.0215 (11)	0.0215 (9)	0.0265 (9)	−0.0042 (8)	0.0061 (8)	−0.0024 (7)
N2G	0.0217 (11)	0.0216 (9)	0.0255 (9)	−0.0007 (8)	0.0048 (8)	−0.0030 (7)
N3G	0.0174 (10)	0.0173 (8)	0.0212 (9)	0.0000 (7)	0.0054 (7)	−0.0003 (7)
N4G	0.0174 (10)	0.0232 (9)	0.0201 (9)	0.0007 (7)	0.0038 (7)	−0.0008 (7)
C1G	0.0243 (13)	0.0219 (11)	0.0253 (11)	0.0042 (9)	0.0034 (9)	0.0003 (9)
C2G	0.0278 (14)	0.0274 (12)	0.0246 (11)	0.0049 (10)	0.0064 (10)	0.0023 (9)
C3G	0.0264 (14)	0.0338 (13)	0.0223 (11)	0.0092 (10)	0.0065 (10)	−0.0038 (9)
C4G	0.0354 (15)	0.0238 (11)	0.0255 (11)	0.0115 (10)	0.0018 (10)	−0.0027 (9)
C5G	0.0283 (14)	0.0215 (11)	0.0240 (11)	0.0039 (9)	0.0001 (10)	−0.0001 (9)
C6G	0.0193 (12)	0.0231 (11)	0.0194 (10)	0.0037 (9)	−0.0010 (9)	−0.0031 (8)
C7G	0.0222 (13)	0.0206 (11)	0.0216 (11)	0.0000 (9)	0.0007 (9)	−0.0002 (8)
C8G	0.0192 (12)	0.0236 (11)	0.0188 (10)	−0.0012 (9)	0.0016 (9)	0.0007 (8)
C9G	0.0203 (13)	0.0210 (10)	0.0185 (10)	0.0017 (9)	0.0020 (9)	0.0001 (8)
C10G	0.0196 (12)	0.0174 (10)	0.0232 (10)	0.0005 (8)	0.0051 (9)	0.0004 (8)
C11G	0.0201 (12)	0.0142 (10)	0.0257 (11)	−0.0007 (8)	0.0045 (9)	−0.0023 (8)
C12G	0.0230 (13)	0.0247 (11)	0.0254 (11)	−0.0004 (9)	0.0078 (9)	−0.0017 (9)
C13G	0.0328 (15)	0.0270 (12)	0.0232 (11)	−0.0001 (10)	0.0054 (10)	−0.0020 (9)
C14G	0.0265 (14)	0.0173 (10)	0.0340 (13)	0.0008 (9)	−0.0011 (10)	−0.0009 (9)
C15G	0.0190 (13)	0.0254 (12)	0.0407 (13)	−0.0001 (9)	0.0067 (10)	−0.0017 (10)
C16G	0.0237 (13)	0.0228 (11)	0.0275 (11)	−0.0011 (9)	0.0094 (10)	−0.0001 (9)
C17G	0.0332 (15)	0.0323 (13)	0.0448 (15)	0.0038 (11)	−0.0097 (12)	0.0017 (11)
C18G	0.084 (3)	0.0505 (18)	0.061 (2)	0.0240 (17)	−0.0443 (18)	−0.0234 (15)
C19G	0.063 (2)	0.0565 (19)	0.0480 (17)	0.0131 (16)	−0.0270 (15)	−0.0160 (14)
C20G	0.062 (2)	0.0312 (14)	0.0505 (17)	−0.0050 (13)	−0.0091 (15)	−0.0001 (12)
C21G	0.0243 (13)	0.0200 (11)	0.0348 (12)	0.0023 (9)	0.0019 (10)	−0.0024 (9)
Cl1H	0.0265 (3)	0.0524 (4)	0.0345 (3)	0.0095 (3)	0.0128 (3)	−0.0033 (3)
S1H	0.0278 (3)	0.0208 (3)	0.0297 (3)	−0.0036 (2)	0.0114 (2)	−0.0023 (2)
N1H	0.0217 (11)	0.0224 (9)	0.0290 (10)	−0.0044 (8)	0.0107 (8)	−0.0020 (7)
N2H	0.0221 (11)	0.0211 (9)	0.0286 (10)	−0.0011 (8)	0.0069 (8)	−0.0032 (7)
N3H	0.0170 (10)	0.0205 (9)	0.0224 (9)	0.0015 (7)	0.0063 (7)	0.0013 (7)
N4H	0.0178 (10)	0.0231 (9)	0.0210 (9)	0.0013 (7)	0.0054 (7)	0.0001 (7)
C1H	0.0224 (13)	0.0229 (11)	0.0247 (11)	0.0036 (9)	0.0026 (9)	0.0026 (9)
C2H	0.0229 (13)	0.0285 (12)	0.0261 (11)	0.0014 (10)	0.0049 (10)	0.0058 (9)
C3H	0.0188 (12)	0.0364 (13)	0.0175 (10)	0.0076 (10)	0.0021 (9)	−0.0031 (9)
C4H	0.0264 (14)	0.0266 (12)	0.0242 (11)	0.0071 (10)	0.0029 (10)	−0.0039 (9)
C5H	0.0238 (13)	0.0224 (11)	0.0255 (11)	0.0010 (9)	0.0027 (9)	−0.0012 (9)
C6H	0.0170 (12)	0.0232 (11)	0.0182 (10)	0.0012 (9)	0.0011 (8)	−0.0008 (8)
C7H	0.0177 (12)	0.0219 (11)	0.0216 (10)	−0.0003 (9)	0.0024 (9)	0.0023 (8)
C8H	0.0198 (12)	0.0224 (11)	0.0209 (10)	0.0014 (9)	0.0046 (9)	0.0030 (8)
C9H	0.0164 (12)	0.0209 (10)	0.0219 (10)	0.0026 (9)	0.0041 (9)	0.0000 (8)
C10H	0.0176 (12)	0.0209 (10)	0.0255 (11)	0.0007 (9)	0.0061 (9)	0.0024 (8)
C11H	0.0198 (12)	0.0140 (10)	0.0251 (11)	0.0012 (8)	0.0060 (9)	0.0027 (8)
C12H	0.0211 (13)	0.0241 (11)	0.0295 (12)	0.0040 (9)	0.0101 (10)	0.0006 (9)
C13H	0.0314 (14)	0.0226 (11)	0.0231 (11)	0.0060 (10)	0.0098 (10)	−0.0012 (9)
C14H	0.0216 (13)	0.0146 (10)	0.0289 (11)	0.0008 (9)	0.0045 (9)	0.0010 (8)
C15H	0.0164 (12)	0.0256 (11)	0.0306 (12)	0.0009 (9)	0.0092 (9)	0.0031 (9)
C16H	0.0222 (13)	0.0224 (11)	0.0235 (11)	−0.0002 (9)	0.0100 (9)	0.0016 (8)
C17H	0.0270 (14)	0.0237 (11)	0.0276 (12)	−0.0025 (10)	0.0023 (10)	−0.0010 (9)
C18H	0.0279 (14)	0.0261 (11)	0.0239 (11)	0.0063 (10)	0.0079 (10)	0.0001 (9)
C19H	0.0362 (16)	0.0386 (14)	0.0289 (12)	0.0106 (11)	0.0020 (11)	−0.0023 (10)
C20H	0.0391 (16)	0.0269 (12)	0.0331 (13)	0.0069 (11)	0.0136 (11)	0.0057 (10)
C21H	0.0207 (13)	0.0234 (11)	0.0362 (13)	0.0030 (9)	0.0067 (10)	0.0015 (9)
Cl1I	0.0440 (4)	0.0502 (4)	0.0469 (4)	0.0210 (3)	0.0258 (3)	0.0029 (3)
S1I	0.0290 (3)	0.0224 (3)	0.0327 (3)	−0.0040 (2)	0.0125 (3)	−0.0031 (2)
N1I	0.0218 (11)	0.0218 (9)	0.0309 (10)	−0.0042 (8)	0.0104 (8)	−0.0017 (8)
N2I	0.0229 (11)	0.0220 (9)	0.0299 (10)	−0.0022 (8)	0.0085 (8)	−0.0026 (8)
N3I	0.0181 (10)	0.0199 (9)	0.0231 (9)	0.0004 (7)	0.0060 (7)	0.0008 (7)
N4I	0.0192 (10)	0.0228 (9)	0.0228 (9)	0.0014 (8)	0.0072 (8)	−0.0012 (7)
C1I	0.0248 (13)	0.0226 (11)	0.0266 (11)	0.0055 (9)	0.0035 (10)	−0.0004 (9)
C2I	0.0282 (14)	0.0275 (12)	0.0265 (11)	0.0064 (10)	0.0081 (10)	0.0040 (9)
C3I	0.0278 (14)	0.0347 (13)	0.0233 (11)	0.0100 (10)	0.0075 (10)	−0.0014 (9)
C4I	0.0343 (15)	0.0222 (11)	0.0277 (12)	0.0111 (10)	0.0035 (10)	−0.0032 (9)
C5I	0.0278 (14)	0.0220 (11)	0.0250 (11)	0.0035 (9)	0.0022 (10)	−0.0010 (9)
C6I	0.0182 (12)	0.0225 (11)	0.0203 (10)	0.0038 (9)	0.0017 (9)	−0.0022 (8)
C7I	0.0198 (12)	0.0199 (10)	0.0227 (11)	−0.0003 (9)	0.0007 (9)	0.0008 (8)
C8I	0.0204 (13)	0.0251 (11)	0.0228 (11)	0.0004 (9)	0.0052 (9)	0.0027 (9)
C9I	0.0210 (13)	0.0212 (11)	0.0228 (11)	0.0031 (9)	0.0049 (9)	0.0005 (8)
C10I	0.0210 (12)	0.0201 (10)	0.0241 (11)	0.0023 (9)	0.0080 (9)	0.0005 (8)
C11I	0.0203 (12)	0.0135 (9)	0.0253 (11)	0.0003 (8)	0.0069 (9)	0.0030 (8)
C12I	0.0215 (13)	0.0268 (11)	0.0305 (12)	0.0030 (9)	0.0118 (10)	0.0019 (9)
C13I	0.0325 (14)	0.0256 (11)	0.0245 (11)	0.0037 (10)	0.0111 (10)	−0.0017 (9)
C14I	0.0261 (13)	0.0150 (10)	0.0292 (11)	−0.0001 (9)	0.0075 (10)	0.0027 (8)
C15I	0.0194 (13)	0.0282 (12)	0.0329 (12)	0.0023 (9)	0.0096 (10)	0.0043 (9)
C16I	0.0252 (13)	0.0249 (11)	0.0246 (11)	0.0029 (9)	0.0117 (9)	0.0024 (9)
C17I	0.0311 (14)	0.0246 (11)	0.0267 (12)	−0.0029 (10)	0.0019 (10)	−0.0001 (9)
C18I	0.0328 (14)	0.0281 (12)	0.0232 (11)	0.0076 (10)	0.0100 (10)	0.0020 (9)
C19I	0.0388 (16)	0.0445 (15)	0.0298 (13)	0.0106 (12)	0.0041 (11)	0.0018 (11)
C20I	0.0454 (17)	0.0281 (12)	0.0353 (13)	0.0103 (11)	0.0174 (12)	0.0073 (10)
C21I	0.0223 (13)	0.0226 (11)	0.0316 (12)	0.0047 (9)	0.0069 (10)	0.0003 (9)

### Geometric parameters (Å, °)

**Table:**

Cl1A—C3A	1.745 (2)	C6E—C7E	1.463 (3)
S1A—C8A	1.689 (2)	C7E—H7EA	0.93
N1A—C8A	1.333 (3)	C9E—C10E	1.499 (3)
N1A—N2A	1.376 (2)	C10E—C11E	1.523 (3)
N1A—H1AB	0.86	C10E—C21E	1.529 (3)
N2A—C9A	1.297 (3)	C10E—H10E	0.98
N3A—C8A	1.383 (3)	C11E—C16E	1.388 (3)
N3A—C9A	1.386 (3)	C11E—C12E	1.398 (3)
N3A—N4A	1.396 (2)	C12E—C13E	1.381 (3)
N4A—C7A	1.280 (3)	C12E—H12E	0.93
C1A—C2A	1.375 (3)	C13E—C14E	1.394 (3)
C1A—C6A	1.401 (3)	C13E—H13E	0.93
C1A—H1AA	0.93	C14E—C15E	1.394 (3)
C2A—C3A	1.390 (3)	C14E—C17E	1.508 (3)
C2A—H2AA	0.93	C15E—C16E	1.387 (3)
C3A—C4A	1.381 (3)	C15E—H15E	0.93
C4A—C5A	1.385 (3)	C16E—H16E	0.93
C4A—H4AA	0.93	C17E—C18E	1.537 (3)
C5A—C6A	1.399 (3)	C17E—H17H	0.97
C5A—H5AA	0.93	C17E—H17I	0.97
C6A—C7A	1.458 (3)	C18E—C20E	1.518 (3)
C7A—H7AA	0.93	C18E—C19E	1.528 (3)
C9A—C10A	1.503 (3)	C18E—H18E	0.98
C10A—C11A	1.525 (3)	C19E—H19P	0.96
C10A—C21A	1.532 (3)	C19E—H19Q	0.96
C10A—H10A	0.98	C19E—H19R	0.96
C11A—C16A	1.392 (3)	C20E—H20P	0.96
C11A—C12A	1.399 (3)	C20E—H20Q	0.96
C12A—C13A	1.384 (3)	C20E—H20R	0.96
C12A—H12A	0.93	C21E—H21M	0.96
C13A—C14A	1.389 (3)	C21E—H21N	0.96
C13A—H13A	0.93	C21E—H21O	0.96
C14A—C15A	1.393 (3)	Cl1F—C3F	1.737 (2)
C14A—C17A	1.512 (3)	S1F—C8F	1.686 (2)
C15A—C16A	1.382 (3)	N1F—C8F	1.335 (3)
C15A—H15A	0.93	N1F—N2F	1.376 (2)
C16A—H16A	0.93	N1F—H1FB	0.86
C17A—C18A	1.508 (3)	N2F—C9F	1.294 (3)
C17A—H17A	0.97	N3F—C8F	1.384 (3)
C17A—H17B	0.97	N3F—C9F	1.388 (3)
C18A—C20A	1.472 (4)	N3F—N4F	1.395 (2)
C18A—C19A	1.538 (3)	N4F—C7F	1.281 (3)
C18A—H18A	0.98	C1F—C2F	1.381 (3)
C19A—H19A	0.96	C1F—C6F	1.397 (3)
C19A—H19B	0.96	C1F—H1FA	0.93
C19A—H19C	0.96	C2F—C3F	1.389 (3)
C20A—H20A	0.96	C2F—H2FA	0.93
C20A—H20B	0.96	C3F—C4F	1.384 (3)
C20A—H20C	0.96	C4F—C5F	1.378 (3)
C21A—H21A	0.96	C4F—H4FA	0.93
C21A—H21B	0.96	C5F—C6F	1.394 (3)
C21A—H21C	0.96	C5F—H5FA	0.93
Cl1B—C3B	1.742 (2)	C6F—C7F	1.461 (3)
S1B—C8B	1.684 (2)	C7F—H7FA	0.93
N1B—C8B	1.338 (3)	C9F—C10F	1.501 (3)
N1B—N2B	1.383 (2)	C10F—C11F	1.521 (3)
N1B—H1BB	0.86	C10F—C21F	1.534 (3)
N2B—C9B	1.293 (3)	C10F—H10F	0.98
N3B—C8B	1.386 (3)	C11F—C16F	1.391 (3)
N3B—C9B	1.387 (3)	C11F—C12F	1.392 (3)
N3B—N4B	1.394 (2)	C12F—C13F	1.383 (3)
N4B—C7B	1.283 (3)	C12F—H12F	0.93
C1B—C2B	1.379 (3)	C13F—C14F	1.390 (3)
C1B—C6B	1.395 (3)	C13F—H13F	0.93
C1B—H1BA	0.93	C14F—C15F	1.397 (3)
C2B—C3B	1.390 (3)	C14F—C17F	1.505 (3)
C2B—H2BA	0.93	C15F—C16F	1.383 (3)
C3B—C4B	1.379 (3)	C15F—H15F	0.93
C4B—C5B	1.376 (3)	C16F—H16F	0.93
C4B—H4BA	0.93	C17F—C18F	1.537 (3)
C5B—C6B	1.399 (3)	C17F—H17J	0.97
C5B—H5BA	0.93	C17F—H17K	0.97
C6B—C7B	1.461 (3)	C18F—C20F	1.513 (3)
C7B—H7BA	0.93	C18F—C19F	1.525 (3)
C9B—C10B	1.500 (3)	C18F—H18F	0.98
C10B—C11B	1.521 (3)	C19F—H19S	0.96
C10B—C21B	1.528 (3)	C19F—H19T	0.96
C10B—H10B	0.98	C19F—H19U	0.96
C11B—C16B	1.387 (3)	C20F—H20S	0.96
C11B—C12B	1.390 (3)	C20F—H20T	0.96
C12B—C13B	1.387 (3)	C20F—H20U	0.96
C12B—H12B	0.93	C21F—H21P	0.96
C13B—C14B	1.388 (3)	C21F—H21Q	0.96
C13B—H13B	0.93	C21F—H21R	0.96
C14B—C15B	1.396 (3)	Cl1G—C3G	1.743 (2)
C14B—C17B	1.504 (3)	S1G—C8G	1.684 (2)
C15B—C16B	1.392 (3)	N1G—C8G	1.338 (3)
C15B—H15B	0.93	N1G—N2G	1.375 (2)
C16B—H16B	0.93	N1G—H1GB	0.86
C17B—C18B	1.509 (4)	N2G—C9G	1.292 (3)
C17B—C18X	1.532 (9)	N3G—C8G	1.387 (3)
C17B—H17C	0.97	N3G—C9G	1.388 (2)
C17B—H17Q	0.97	N3G—N4G	1.397 (2)
C17B—H17R	0.96	N4G—C7G	1.284 (3)
C17B—H17S	0.96	C1G—C2G	1.375 (3)
C18B—C19B	1.499 (6)	C1G—C6G	1.402 (3)
C18B—C20B	1.523 (5)	C1G—H1GA	0.93
C18B—H18B	0.98	C2G—C3G	1.391 (3)
C19B—H19D	0.96	C2G—H2GA	0.93
C19B—H19E	0.96	C3G—C4G	1.380 (3)
C19B—H19F	0.96	C4G—C5G	1.380 (3)
C20B—H20D	0.96	C4G—H4GA	0.93
C20B—H20E	0.96	C5G—C6G	1.400 (3)
C20B—H20F	0.96	C5G—H5GA	0.93
C18X—C19X	1.16 (2)	C6G—C7G	1.461 (3)
C18X—C20X	1.591 (17)	C7G—H7GA	0.93
C18X—H18J	0.98	C9G—C10G	1.502 (3)
C19X—H19G	0.96	C10G—C11G	1.518 (3)
C19X—H19H	0.96	C10G—C21G	1.535 (3)
C19X—H19I	0.96	C10G—H10G	0.98
C20X—H20G	0.96	C11G—C16G	1.391 (3)
C20X—H20H	0.96	C11G—C12G	1.393 (3)
C20X—H20I	0.96	C12G—C13G	1.389 (3)
C21B—H21D	0.96	C12G—H12G	0.93
C21B—H21E	0.96	C13G—C14G	1.391 (3)
C21B—H21F	0.96	C13G—H13G	0.93
Cl1C—C3C	1.742 (2)	C14G—C15G	1.389 (3)
S1C—C8C	1.685 (2)	C14G—C17G	1.511 (3)
N1C—C8C	1.338 (3)	C15G—C16G	1.389 (3)
N1C—N2C	1.379 (2)	C15G—H15G	0.93
N1C—H1CB	0.86	C16G—H16G	0.93
N2C—C9C	1.295 (3)	C17G—C18G	1.466 (4)
N3C—C8C	1.384 (3)	C17G—H17L	0.97
N3C—C9C	1.386 (3)	C17G—H17M	0.97
N3C—N4C	1.393 (2)	C18G—C20G	1.440 (4)
N4C—C7C	1.278 (3)	C18G—C19G	1.536 (4)
C1C—C2C	1.382 (3)	C18G—H18G	0.98
C1C—C6C	1.399 (3)	C19G—H19V	0.96
C1C—H1CA	0.93	C19G—H19W	0.96
C2C—C3C	1.386 (3)	C19G—H19X	0.96
C2C—H2CA	0.93	C20G—H20V	0.96
C3C—C4C	1.382 (3)	C20G—H20W	0.96
C4C—C5C	1.376 (3)	C20G—H20X	0.96
C4C—H4CA	0.93	C21G—H21S	0.96
C5C—C6C	1.394 (3)	C21G—H21T	0.96
C5C—H5CA	0.93	C21G—H21U	0.96
C6C—C7C	1.464 (3)	Cl1H—C3H	1.747 (2)
C7C—H7CA	0.93	S1H—C8H	1.685 (2)
C9C—C10C	1.498 (3)	N1H—C8H	1.334 (3)
C10C—C11C	1.519 (3)	N1H—N2H	1.379 (2)
C10C—C21C	1.530 (3)	N1H—H1HB	0.86
C10C—H10C	0.98	N2H—C9H	1.297 (3)
C11C—C16C	1.388 (3)	N3H—C8H	1.383 (3)
C11C—C12C	1.393 (3)	N3H—C9H	1.386 (2)
C12C—C13C	1.381 (3)	N3H—N4H	1.399 (2)
C12C—H12C	0.93	N4H—C7H	1.280 (3)
C13C—C14C	1.389 (3)	C1H—C2H	1.380 (3)
C13C—H13C	0.93	C1H—C6H	1.398 (3)
C14C—C15C	1.398 (3)	C1H—H1HA	0.93
C14C—C17C	1.515 (3)	C2H—C3H	1.390 (3)
C15C—C16C	1.387 (3)	C2H—H2HA	0.93
C15C—H15C	0.93	C3H—C4H	1.380 (3)
C16C—H16C	0.93	C4H—C5H	1.382 (3)
C17C—C18C	1.522 (3)	C4H—H4HA	0.93
C17C—H17D	0.97	C5H—C6H	1.401 (3)
C17C—H17E	0.97	C5H—H5HA	0.93
C18C—C19C	1.490 (3)	C6H—C7H	1.456 (3)
C18C—C20C	1.540 (3)	C7H—H7HA	0.93
C18C—H18C	0.98	C9H—C10H	1.498 (3)
C19C—H19J	0.96	C10H—C11H	1.522 (3)
C19C—H19K	0.96	C10H—C21H	1.533 (3)
C19C—H19L	0.96	C10H—H10H	0.98
C20C—H20J	0.96	C11H—C12H	1.394 (3)
C20C—H20K	0.96	C11H—C16H	1.394 (3)
C20C—H20L	0.96	C12H—C13H	1.386 (3)
C21C—H21G	0.96	C12H—H12H	0.93
C21C—H21H	0.96	C13H—C14H	1.389 (3)
C21C—H21I	0.96	C13H—H13H	0.93
Cl1D—C3D	1.744 (2)	C14H—C15H	1.394 (3)
S1D—C8D	1.688 (2)	C14H—C17H	1.508 (3)
N1D—C8D	1.331 (3)	C15H—C16H	1.381 (3)
N1D—N2D	1.377 (2)	C15H—H15H	0.93
N1D—H1DB	0.86	C16H—H16H	0.93
N2D—C9D	1.294 (3)	C17H—C18H	1.532 (3)
N3D—C8D	1.382 (3)	C17H—H17N	0.97
N3D—C9D	1.388 (2)	C17H—H17O	0.97
N3D—N4D	1.400 (2)	C18H—C20H	1.524 (3)
N4D—C7D	1.280 (3)	C18H—C19H	1.527 (3)
C1D—C2D	1.379 (3)	C18H—H18H	0.98
C1D—C6D	1.398 (3)	C19H—H19Y	0.96
C1D—H1DA	0.93	C19H—H19Z	0.96
C2D—C3D	1.386 (3)	C19H—H191	0.96
C2D—H2DA	0.93	C20H—H20Y	0.96
C3D—C4D	1.382 (3)	C20H—H20Z	0.96
C4D—C5D	1.379 (3)	C20H—H21V	0.96
C4D—H4DA	0.93	C21H—H21W	0.96
C5D—C6D	1.401 (3)	C21H—H21X	0.96
C5D—H5DA	0.93	C21H—H21Y	0.96
C6D—C7D	1.457 (3)	Cl1I—C3I	1.743 (2)
C7D—H7DA	0.93	S1I—C8I	1.683 (2)
C9D—C10D	1.499 (3)	N1I—C8I	1.334 (3)
C10D—C11D	1.521 (3)	N1I—N2I	1.376 (2)
C10D—C21D	1.526 (3)	N1I—H1IB	0.86
C10D—H10D	0.98	N2I—C9I	1.299 (3)
C11D—C16D	1.387 (3)	N3I—C8I	1.384 (3)
C11D—C12D	1.392 (3)	N3I—C9I	1.390 (3)
C12D—C13D	1.384 (3)	N3I—N4I	1.399 (2)
C12D—H12D	0.93	N4I—C7I	1.285 (3)
C13D—C14D	1.392 (3)	C1I—C2I	1.381 (3)
C13D—H13D	0.93	C1I—C6I	1.398 (3)
C14D—C15D	1.386 (3)	C1I—H1IA	0.93
C14D—C17D	1.507 (3)	C2I—C3I	1.391 (3)
C15D—C16D	1.388 (3)	C2I—H2IA	0.93
C15D—H15D	0.93	C3I—C4I	1.381 (3)
C16D—H16D	0.93	C4I—C5I	1.380 (3)
C17D—C18D	1.508 (3)	C4I—H4IA	0.93
C17D—H17F	0.97	C5I—C6I	1.403 (3)
C17D—H17G	0.97	C5I—H5IA	0.93
C18D—C20D	1.491 (3)	C6I—C7I	1.458 (3)
C18D—C19D	1.530 (3)	C7I—H7IA	0.93
C18D—H18D	0.98	C9I—C10I	1.499 (3)
C19D—H19M	0.96	C10I—C11I	1.521 (3)
C19D—H19N	0.96	C10I—C21I	1.533 (3)
C19D—H19O	0.96	C10I—H10I	0.98
C20D—H20M	0.96	C11I—C16I	1.393 (3)
C20D—H20N	0.96	C11I—C12I	1.394 (3)
C20D—H20O	0.96	C12I—C13I	1.386 (3)
C21D—H21J	0.96	C12I—H12I	0.93
C21D—H21K	0.96	C13I—C14I	1.387 (3)
C21D—H21L	0.96	C13I—H13I	0.93
Cl1E—C3E	1.742 (2)	C14I—C15I	1.394 (3)
S1E—C8E	1.686 (2)	C14I—C17I	1.508 (3)
N1E—C8E	1.339 (3)	C15I—C16I	1.384 (3)
N1E—N2E	1.376 (2)	C15I—H15I	0.93
N1E—H1EB	0.86	C16I—H16I	0.93
N2E—C9E	1.292 (3)	C17I—C18I	1.533 (3)
N3E—C8E	1.381 (3)	C17I—H17T	0.97
N3E—C9E	1.388 (2)	C17I—H17U	0.97
N3E—N4E	1.393 (2)	C18I—C20I	1.517 (3)
N4E—C7E	1.281 (3)	C18I—C19I	1.528 (3)
C1E—C2E	1.382 (3)	C18I—H18I	0.98
C1E—C6E	1.393 (3)	C19I—H192	0.96
C1E—H1EA	0.93	C19I—H193	0.96
C2E—C3E	1.390 (3)	C19I—H194	0.96
C2E—H2EA	0.93	C20I—H21Z	0.96
C3E—C4E	1.373 (3)	C20I—H22A	0.96
C4E—C5E	1.382 (3)	C20I—H22B	0.96
C4E—H4EA	0.93	C21I—H22C	0.96
C5E—C6E	1.397 (3)	C21I—H22D	0.96
C5E—H5EA	0.93	C21I—H22E	0.96
			
C8A—N1A—N2A	114.17 (18)	C9E—C10E—C11E	109.83 (16)
C8A—N1A—H1AB	122.9	C9E—C10E—C21E	109.97 (18)
N2A—N1A—H1AB	122.9	C11E—C10E—C21E	112.18 (16)
C9A—N2A—N1A	104.14 (17)	C9E—C10E—H10E	108.3
C8A—N3A—C9A	107.86 (17)	C11E—C10E—H10E	108.3
C8A—N3A—N4A	130.26 (16)	C21E—C10E—H10E	108.3
C9A—N3A—N4A	121.09 (17)	C16E—C11E—C12E	118.05 (19)
C7A—N4A—N3A	115.75 (17)	C16E—C11E—C10E	121.86 (18)
C2A—C1A—C6A	120.8 (2)	C12E—C11E—C10E	120.09 (19)
C2A—C1A—H1AA	119.6	C13E—C12E—C11E	120.7 (2)
C6A—C1A—H1AA	119.6	C13E—C12E—H12E	119.7
C1A—C2A—C3A	119.0 (2)	C11E—C12E—H12E	119.7
C1A—C2A—H2AA	120.5	C12E—C13E—C14E	121.6 (2)
C3A—C2A—H2AA	120.5	C12E—C13E—H13E	119.2
C4A—C3A—C2A	121.9 (2)	C14E—C13E—H13E	119.2
C4A—C3A—Cl1A	119.05 (17)	C13E—C14E—C15E	117.4 (2)
C2A—C3A—Cl1A	119.06 (18)	C13E—C14E—C17E	120.9 (2)
C3A—C4A—C5A	118.5 (2)	C15E—C14E—C17E	121.7 (2)
C3A—C4A—H4AA	120.7	C16E—C15E—C14E	121.2 (2)
C5A—C4A—H4AA	120.7	C16E—C15E—H15E	119.4
C4A—C5A—C6A	121.1 (2)	C14E—C15E—H15E	119.4
C4A—C5A—H5AA	119.4	C15E—C16E—C11E	121.0 (2)
C6A—C5A—H5AA	119.4	C15E—C16E—H16E	119.5
C5A—C6A—C1A	118.6 (2)	C11E—C16E—H16E	119.5
C5A—C6A—C7A	118.84 (19)	C14E—C17E—C18E	114.69 (17)
C1A—C6A—C7A	122.52 (19)	C14E—C17E—H17H	108.6
N4A—C7A—C6A	120.83 (19)	C18E—C17E—H17H	108.6
N4A—C7A—H7AA	119.6	C14E—C17E—H17I	108.6
C6A—C7A—H7AA	119.6	C18E—C17E—H17I	108.6
N1A—C8A—N3A	102.95 (17)	H17H—C17E—H17I	107.6
N1A—C8A—S1A	127.91 (16)	C20E—C18E—C19E	110.29 (19)
N3A—C8A—S1A	129.03 (16)	C20E—C18E—C17E	111.94 (19)
N2A—C9A—N3A	110.79 (18)	C19E—C18E—C17E	109.42 (18)
N2A—C9A—C10A	126.12 (19)	C20E—C18E—H18E	108.4
N3A—C9A—C10A	123.07 (19)	C19E—C18E—H18E	108.4
C9A—C10A—C11A	112.02 (17)	C17E—C18E—H18E	108.4
C9A—C10A—C21A	110.50 (18)	C18E—C19E—H19P	109.5
C11A—C10A—C21A	111.09 (16)	C18E—C19E—H19Q	109.5
C9A—C10A—H10A	107.7	H19P—C19E—H19Q	109.5
C11A—C10A—H10A	107.7	C18E—C19E—H19R	109.5
C21A—C10A—H10A	107.7	H19P—C19E—H19R	109.5
C16A—C11A—C12A	118.2 (2)	H19Q—C19E—H19R	109.5
C16A—C11A—C10A	120.83 (19)	C18E—C20E—H20P	109.5
C12A—C11A—C10A	120.9 (2)	C18E—C20E—H20Q	109.5
C13A—C12A—C11A	120.2 (2)	H20P—C20E—H20Q	109.5
C13A—C12A—H12A	119.9	C18E—C20E—H20R	109.5
C11A—C12A—H12A	119.9	H20P—C20E—H20R	109.5
C12A—C13A—C14A	121.7 (2)	H20Q—C20E—H20R	109.5
C12A—C13A—H13A	119.1	C10E—C21E—H21M	109.5
C14A—C13A—H13A	119.1	C10E—C21E—H21N	109.5
C13A—C14A—C15A	117.6 (2)	H21M—C21E—H21N	109.5
C13A—C14A—C17A	120.9 (2)	C10E—C21E—H21O	109.5
C15A—C14A—C17A	121.5 (2)	H21M—C21E—H21O	109.5
C16A—C15A—C14A	121.3 (2)	H21N—C21E—H21O	109.5
C16A—C15A—H15A	119.4	C8F—N1F—N2F	114.33 (17)
C14A—C15A—H15A	119.4	C8F—N1F—H1FB	122.8
C15A—C16A—C11A	120.9 (2)	N2F—N1F—H1FB	122.8
C15A—C16A—H16A	119.5	C9F—N2F—N1F	104.13 (17)
C11A—C16A—H16A	119.5	C8F—N3F—C9F	107.97 (17)
C18A—C17A—C14A	114.17 (19)	C8F—N3F—N4F	132.49 (16)
C18A—C17A—H17A	108.7	C9F—N3F—N4F	119.16 (16)
C14A—C17A—H17A	108.7	C7F—N4F—N3F	117.26 (17)
C18A—C17A—H17B	108.7	C2F—C1F—C6F	120.9 (2)
C14A—C17A—H17B	108.7	C2F—C1F—H1FA	119.5
H17A—C17A—H17B	107.6	C6F—C1F—H1FA	119.5
C20A—C18A—C17A	115.0 (2)	C1F—C2F—C3F	118.9 (2)
C20A—C18A—C19A	110.7 (2)	C1F—C2F—H2FA	120.5
C17A—C18A—C19A	110.4 (2)	C3F—C2F—H2FA	120.5
C20A—C18A—H18A	106.7	C4F—C3F—C2F	121.4 (2)
C17A—C18A—H18A	106.7	C4F—C3F—Cl1F	119.17 (18)
C19A—C18A—H18A	106.7	C2F—C3F—Cl1F	119.44 (18)
C18A—C19A—H19A	109.5	C5F—C4F—C3F	118.9 (2)
C18A—C19A—H19B	109.5	C5F—C4F—H4FA	120.5
H19A—C19A—H19B	109.5	C3F—C4F—H4FA	120.5
C18A—C19A—H19C	109.5	C4F—C5F—C6F	121.3 (2)
H19A—C19A—H19C	109.5	C4F—C5F—H5FA	119.4
H19B—C19A—H19C	109.5	C6F—C5F—H5FA	119.4
C18A—C20A—H20A	109.5	C5F—C6F—C1F	118.6 (2)
C18A—C20A—H20B	109.5	C5F—C6F—C7F	118.79 (19)
H20A—C20A—H20B	109.5	C1F—C6F—C7F	122.60 (19)
C18A—C20A—H20C	109.5	N4F—C7F—C6F	119.14 (19)
H20A—C20A—H20C	109.5	N4F—C7F—H7FA	120.4
H20B—C20A—H20C	109.5	C6F—C7F—H7FA	120.4
C10A—C21A—H21A	109.5	N1F—C8F—N3F	102.68 (17)
C10A—C21A—H21B	109.5	N1F—C8F—S1F	126.67 (16)
H21A—C21A—H21B	109.5	N3F—C8F—S1F	130.59 (16)
C10A—C21A—H21C	109.5	N2F—C9F—N3F	110.81 (18)
H21A—C21A—H21C	109.5	N2F—C9F—C10F	125.90 (19)
H21B—C21A—H21C	109.5	N3F—C9F—C10F	123.29 (19)
C8B—N1B—N2B	114.07 (17)	C9F—C10F—C11F	111.67 (16)
C8B—N1B—H1BB	123.0	C9F—C10F—C21F	109.44 (18)
N2B—N1B—H1BB	123.0	C11F—C10F—C21F	111.37 (16)
C9B—N2B—N1B	104.32 (17)	C9F—C10F—H10F	108.1
C8B—N3B—C9B	108.34 (17)	C11F—C10F—H10F	108.1
C8B—N3B—N4B	132.47 (17)	C21F—C10F—H10F	108.1
C9B—N3B—N4B	118.85 (16)	C16F—C11F—C12F	118.24 (19)
C7B—N4B—N3B	118.20 (17)	C16F—C11F—C10F	121.56 (18)
C2B—C1B—C6B	120.9 (2)	C12F—C11F—C10F	120.14 (19)
C2B—C1B—H1BA	119.6	C13F—C12F—C11F	120.6 (2)
C6B—C1B—H1BA	119.6	C13F—C12F—H12F	119.7
C1B—C2B—C3B	119.1 (2)	C11F—C12F—H12F	119.7
C1B—C2B—H2BA	120.4	C12F—C13F—C14F	121.7 (2)
C3B—C2B—H2BA	120.4	C12F—C13F—H13F	119.2
C4B—C3B—C2B	121.1 (2)	C14F—C13F—H13F	119.2
C4B—C3B—Cl1B	119.35 (18)	C13F—C14F—C15F	117.27 (19)
C2B—C3B—Cl1B	119.54 (18)	C13F—C14F—C17F	120.96 (19)
C5B—C4B—C3B	119.5 (2)	C15F—C14F—C17F	121.8 (2)
C5B—C4B—H4BA	120.3	C16F—C15F—C14F	121.4 (2)
C3B—C4B—H4BA	120.3	C16F—C15F—H15F	119.3
C4B—C5B—C6B	120.8 (2)	C14F—C15F—H15F	119.3
C4B—C5B—H5BA	119.6	C15F—C16F—C11F	120.7 (2)
C6B—C5B—H5BA	119.6	C15F—C16F—H16F	119.6
C1B—C6B—C5B	118.6 (2)	C11F—C16F—H16F	119.6
C1B—C6B—C7B	122.04 (19)	C14F—C17F—C18F	114.48 (18)
C5B—C6B—C7B	119.28 (19)	C14F—C17F—H17J	108.6
N4B—C7B—C6B	118.94 (19)	C18F—C17F—H17J	108.6
N4B—C7B—H7BA	120.5	C14F—C17F—H17K	108.6
C6B—C7B—H7BA	120.5	C18F—C17F—H17K	108.6
N1B—C8B—N3B	102.51 (17)	H17J—C17F—H17K	107.6
N1B—C8B—S1B	127.01 (16)	C20F—C18F—C19F	110.59 (19)
N3B—C8B—S1B	130.43 (17)	C20F—C18F—C17F	111.55 (19)
N2B—C9B—N3B	110.67 (18)	C19F—C18F—C17F	110.11 (19)
N2B—C9B—C10B	125.86 (19)	C20F—C18F—H18F	108.2
N3B—C9B—C10B	123.46 (19)	C19F—C18F—H18F	108.2
C9B—C10B—C11B	110.61 (17)	C17F—C18F—H18F	108.2
C9B—C10B—C21B	110.03 (18)	C18F—C19F—H19S	109.5
C11B—C10B—C21B	111.56 (17)	C18F—C19F—H19T	109.5
C9B—C10B—H10B	108.2	H19S—C19F—H19T	109.5
C11B—C10B—H10B	108.2	C18F—C19F—H19U	109.5
C21B—C10B—H10B	108.2	H19S—C19F—H19U	109.5
C16B—C11B—C12B	118.2 (2)	H19T—C19F—H19U	109.5
C16B—C11B—C10B	121.04 (19)	C18F—C20F—H20S	109.5
C12B—C11B—C10B	120.7 (2)	C18F—C20F—H20T	109.5
C13B—C12B—C11B	120.8 (2)	H20S—C20F—H20T	109.5
C13B—C12B—H12B	119.6	C18F—C20F—H20U	109.5
C11B—C12B—H12B	119.6	H20S—C20F—H20U	109.5
C12B—C13B—C14B	121.3 (2)	H20T—C20F—H20U	109.5
C12B—C13B—H13B	119.3	C10F—C21F—H21P	109.5
C14B—C13B—H13B	119.3	C10F—C21F—H21Q	109.5
C13B—C14B—C15B	118.0 (2)	H21P—C21F—H21Q	109.5
C13B—C14B—C17B	120.5 (2)	C10F—C21F—H21R	109.5
C15B—C14B—C17B	121.5 (2)	H21P—C21F—H21R	109.5
C16B—C15B—C14B	120.6 (2)	H21Q—C21F—H21R	109.5
C16B—C15B—H15B	119.7	C8G—N1G—N2G	114.40 (17)
C14B—C15B—H15B	119.7	C8G—N1G—H1GB	122.8
C11B—C16B—C15B	121.2 (2)	N2G—N1G—H1GB	122.8
C11B—C16B—H16B	119.4	C9G—N2G—N1G	104.05 (17)
C15B—C16B—H16B	119.4	C8G—N3G—C9G	107.77 (17)
C14B—C17B—C18B	113.5 (2)	C8G—N3G—N4G	129.69 (16)
C14B—C17B—C18X	116.7 (4)	C9G—N3G—N4G	121.51 (16)
C14B—C17B—H17C	108.9	C7G—N4G—N3G	115.11 (17)
C18B—C17B—H17C	108.9	C2G—C1G—C6G	120.5 (2)
C18X—C17B—H17C	130.9	C2G—C1G—H1GA	119.7
C14B—C17B—H17Q	108.9	C6G—C1G—H1GA	119.7
C18B—C17B—H17Q	108.9	C1G—C2G—C3G	119.2 (2)
C18X—C17B—H17Q	74.6	C1G—C2G—H2GA	120.4
H17C—C17B—H17Q	107.7	C3G—C2G—H2GA	120.4
C14B—C17B—H17R	108.2	C4G—C3G—C2G	121.6 (2)
C18X—C17B—H17R	107.2	C4G—C3G—Cl1G	119.35 (17)
H17Q—C17B—H17R	136.7	C2G—C3G—Cl1G	119.04 (18)
C14B—C17B—H17S	108.0	C5G—C4G—C3G	118.8 (2)
C18B—C17B—H17S	135.2	C5G—C4G—H4GA	120.6
C18X—C17B—H17S	109.0	C3G—C4G—H4GA	120.6
H17C—C17B—H17S	71.3	C4G—C5G—C6G	121.1 (2)
H17R—C17B—H17S	107.3	C4G—C5G—H5GA	119.4
C19B—C18B—C17B	117.3 (3)	C6G—C5G—H5GA	119.4
C19B—C18B—C20B	111.4 (4)	C5G—C6G—C1G	118.7 (2)
C17B—C18B—C20B	111.3 (3)	C5G—C6G—C7G	118.67 (19)
C19B—C18B—H17R	145.3	C1G—C6G—C7G	122.63 (19)
C20B—C18B—H17R	101.8	N4G—C7G—C6G	120.40 (19)
C19B—C18B—H18B	105.2	N4G—C7G—H7GA	119.8
C17B—C18B—H18B	105.2	C6G—C7G—H7GA	119.8
C20B—C18B—H18B	105.2	N1G—C8G—N3G	102.59 (17)
H17R—C18B—H18B	74.4	N1G—C8G—S1G	127.49 (16)
C19X—C18X—C17B	122.8 (13)	N3G—C8G—S1G	129.83 (16)
C19X—C18X—C20X	120.4 (14)	N2G—C9G—N3G	111.14 (18)
C17B—C18X—C20X	109.0 (8)	N2G—C9G—C10G	125.57 (18)
C19X—C18X—H18J	99.4	N3G—C9G—C10G	123.28 (19)
C17B—C18X—H18J	99.4	C9G—C10G—C11G	111.04 (16)
C20X—C18X—H18J	99.4	C9G—C10G—C21G	110.10 (17)
C18X—C19X—H19G	109.5	C11G—C10G—C21G	111.67 (16)
C18X—C19X—H19H	109.5	C9G—C10G—H10G	108.0
H19G—C19X—H19H	109.5	C11G—C10G—H10G	108.0
C18X—C19X—H19I	109.5	C21G—C10G—H10G	108.0
H19G—C19X—H19I	109.5	C16G—C11G—C12G	118.2 (2)
H19H—C19X—H19I	109.5	C16G—C11G—C10G	120.99 (19)
C18X—C20X—H20G	109.5	C12G—C11G—C10G	120.83 (19)
C18X—C20X—H20H	109.5	C13G—C12G—C11G	120.6 (2)
H20G—C20X—H20H	109.5	C13G—C12G—H12G	119.7
C18X—C20X—H20I	109.5	C11G—C12G—H12G	119.7
H20G—C20X—H20I	109.5	C12G—C13G—C14G	121.3 (2)
H20H—C20X—H20I	109.5	C12G—C13G—H13G	119.4
C10B—C21B—H21D	109.5	C14G—C13G—H13G	119.4
C10B—C21B—H21E	109.5	C15G—C14G—C13G	117.9 (2)
H21D—C21B—H21E	109.5	C15G—C14G—C17G	121.9 (2)
C10B—C21B—H21F	109.5	C13G—C14G—C17G	120.2 (2)
H21D—C21B—H21F	109.5	C14G—C15G—C16G	121.1 (2)
H21E—C21B—H21F	109.5	C14G—C15G—H15G	119.5
C8C—N1C—N2C	114.32 (17)	C16G—C15G—H15G	119.5
C8C—N1C—H1CB	122.8	C15G—C16G—C11G	120.9 (2)
N2C—N1C—H1CB	122.8	C15G—C16G—H16G	119.5
C9C—N2C—N1C	103.96 (17)	C11G—C16G—H16G	119.5
C8C—N3C—C9C	108.14 (17)	C18G—C17G—C14G	117.2 (2)
C8C—N3C—N4C	132.89 (17)	C18G—C17G—H17L	108.0
C9C—N3C—N4C	118.64 (16)	C14G—C17G—H17L	108.0
C7C—N4C—N3C	118.28 (17)	C18G—C17G—H17M	108.0
C2C—C1C—C6C	120.6 (2)	C14G—C17G—H17M	108.0
C2C—C1C—H1CA	119.7	H17L—C17G—H17M	107.2
C6C—C1C—H1CA	119.7	C20G—C18G—C17G	120.7 (3)
C1C—C2C—C3C	119.1 (2)	C20G—C18G—C19G	113.0 (2)
C1C—C2C—H2CA	120.4	C17G—C18G—C19G	111.2 (2)
C3C—C2C—H2CA	120.4	C20G—C18G—H18G	103.1
C4C—C3C—C2C	121.3 (2)	C17G—C18G—H18G	103.1
C4C—C3C—Cl1C	119.15 (18)	C19G—C18G—H18G	103.1
C2C—C3C—Cl1C	119.52 (18)	C18G—C19G—H19V	109.5
C5C—C4C—C3C	119.2 (2)	C18G—C19G—H19W	109.5
C5C—C4C—H4CA	120.4	H19V—C19G—H19W	109.5
C3C—C4C—H4CA	120.4	C18G—C19G—H19X	109.5
C4C—C5C—C6C	121.0 (2)	H19V—C19G—H19X	109.5
C4C—C5C—H5CA	119.5	H19W—C19G—H19X	109.5
C6C—C5C—H5CA	119.5	C18G—C20G—H20V	109.5
C5C—C6C—C1C	118.8 (2)	C18G—C20G—H20W	109.5
C5C—C6C—C7C	119.64 (19)	H20V—C20G—H20W	109.5
C1C—C6C—C7C	121.57 (19)	C18G—C20G—H20X	109.5
N4C—C7C—C6C	118.86 (19)	H20V—C20G—H20X	109.5
N4C—C7C—H7CA	120.6	H20W—C20G—H20X	109.5
C6C—C7C—H7CA	120.6	C10G—C21G—H21S	109.5
N1C—C8C—N3C	102.54 (17)	C10G—C21G—H21T	109.5
N1C—C8C—S1C	126.85 (16)	H21S—C21G—H21T	109.5
N3C—C8C—S1C	130.57 (16)	C10G—C21G—H21U	109.5
N2C—C9C—N3C	110.95 (18)	H21S—C21G—H21U	109.5
N2C—C9C—C10C	125.71 (19)	H21T—C21G—H21U	109.5
N3C—C9C—C10C	123.32 (19)	C8H—N1H—N2H	114.15 (17)
C9C—C10C—C11C	110.89 (17)	C8H—N1H—H1HB	122.9
C9C—C10C—C21C	109.96 (18)	N2H—N1H—H1HB	122.9
C11C—C10C—C21C	111.27 (17)	C9H—N2H—N1H	104.22 (16)
C9C—C10C—H10C	108.2	C8H—N3H—C9H	108.30 (17)
C11C—C10C—H10C	108.2	C8H—N3H—N4H	131.08 (16)
C21C—C10C—H10C	108.2	C9H—N3H—N4H	120.06 (16)
C16C—C11C—C12C	118.2 (2)	C7H—N4H—N3H	115.93 (17)
C16C—C11C—C10C	121.25 (19)	C2H—C1H—C6H	120.5 (2)
C12C—C11C—C10C	120.5 (2)	C2H—C1H—H1HA	119.7
C13C—C12C—C11C	120.8 (2)	C6H—C1H—H1HA	119.7
C13C—C12C—H12C	119.6	C1H—C2H—C3H	119.0 (2)
C11C—C12C—H12C	119.6	C1H—C2H—H2HA	120.5
C12C—C13C—C14C	121.5 (2)	C3H—C2H—H2HA	120.5
C12C—C13C—H13C	119.3	C4H—C3H—C2H	121.9 (2)
C14C—C13C—H13C	119.3	C4H—C3H—Cl1H	118.53 (17)
C13C—C14C—C15C	117.6 (2)	C2H—C3H—Cl1H	119.54 (18)
C13C—C14C—C17C	121.1 (2)	C3H—C4H—C5H	118.6 (2)
C15C—C14C—C17C	121.3 (2)	C3H—C4H—H4HA	120.7
C16C—C15C—C14C	121.1 (2)	C5H—C4H—H4HA	120.7
C16C—C15C—H15C	119.5	C4H—C5H—C6H	121.0 (2)
C14C—C15C—H15C	119.5	C4H—C5H—H5HA	119.5
C15C—C16C—C11C	120.9 (2)	C6H—C5H—H5HA	119.5
C15C—C16C—H16C	119.6	C1H—C6H—C5H	118.9 (2)
C11C—C16C—H16C	119.6	C1H—C6H—C7H	123.45 (19)
C14C—C17C—C18C	113.1 (2)	C5H—C6H—C7H	117.59 (19)
C14C—C17C—H17D	109.0	N4H—C7H—C6H	120.79 (19)
C18C—C17C—H17D	109.0	N4H—C7H—H7HA	119.6
C14C—C17C—H17E	109.0	C6H—C7H—H7HA	119.6
C18C—C17C—H17E	109.0	N1H—C8H—N3H	102.69 (17)
H17D—C17C—H17E	107.8	N1H—C8H—S1H	127.36 (16)
C19C—C18C—C17C	113.4 (2)	N3H—C8H—S1H	129.88 (16)
C19C—C18C—C20C	111.2 (2)	N2H—C9H—N3H	110.54 (18)
C17C—C18C—C20C	109.5 (2)	N2H—C9H—C10H	126.10 (18)
C19C—C18C—H18C	107.5	N3H—C9H—C10H	123.35 (18)
C17C—C18C—H18C	107.5	C9H—C10H—C11H	110.34 (16)
C20C—C18C—H18C	107.5	C9H—C10H—C21H	110.16 (17)
C18C—C19C—H19J	109.5	C11H—C10H—C21H	112.99 (17)
C18C—C19C—H19K	109.5	C9H—C10H—H10H	107.7
H19J—C19C—H19K	109.5	C11H—C10H—H10H	107.7
C18C—C19C—H19L	109.5	C21H—C10H—H10H	107.7
H19J—C19C—H19L	109.5	C12H—C11H—C16H	118.03 (19)
H19K—C19C—H19L	109.5	C12H—C11H—C10H	120.81 (19)
C18C—C20C—H20J	109.5	C16H—C11H—C10H	121.15 (18)
C18C—C20C—H20K	109.5	C13H—C12H—C11H	120.7 (2)
H20J—C20C—H20K	109.5	C13H—C12H—H12H	119.6
C18C—C20C—H20L	109.5	C11H—C12H—H12H	119.6
H20J—C20C—H20L	109.5	C12H—C13H—C14H	121.5 (2)
H20K—C20C—H20L	109.5	C12H—C13H—H13H	119.3
C10C—C21C—H21G	109.5	C14H—C13H—H13H	119.3
C10C—C21C—H21H	109.5	C13H—C14H—C15H	117.5 (2)
H21G—C21C—H21H	109.5	C13H—C14H—C17H	121.22 (19)
C10C—C21C—H21I	109.5	C15H—C14H—C17H	121.3 (2)
H21G—C21C—H21I	109.5	C16H—C15H—C14H	121.5 (2)
H21H—C21C—H21I	109.5	C16H—C15H—H15H	119.3
C8D—N1D—N2D	113.98 (17)	C14H—C15H—H15H	119.3
C8D—N1D—H1DB	123.0	C15H—C16H—C11H	120.80 (19)
N2D—N1D—H1DB	123.0	C15H—C16H—H16H	119.6
C9D—N2D—N1D	104.35 (17)	C11H—C16H—H16H	119.6
C8D—N3D—C9D	107.92 (17)	C14H—C17H—C18H	113.95 (17)
C8D—N3D—N4D	130.32 (16)	C14H—C17H—H17N	108.8
C9D—N3D—N4D	120.82 (16)	C18H—C17H—H17N	108.8
C7D—N4D—N3D	115.62 (17)	C14H—C17H—H17O	108.8
C2D—C1D—C6D	120.5 (2)	C18H—C17H—H17O	108.8
C2D—C1D—H1DA	119.7	H17N—C17H—H17O	107.7
C6D—C1D—H1DA	119.7	C20H—C18H—C19H	110.51 (19)
C1D—C2D—C3D	119.2 (2)	C20H—C18H—C17H	111.16 (19)
C1D—C2D—H2DA	120.4	C19H—C18H—C17H	110.33 (18)
C3D—C2D—H2DA	120.4	C20H—C18H—H18H	108.2
C4D—C3D—C2D	121.7 (2)	C19H—C18H—H18H	108.2
C4D—C3D—Cl1D	119.10 (17)	C17H—C18H—H18H	108.2
C2D—C3D—Cl1D	119.18 (18)	C18H—C19H—H19Y	109.5
C5D—C4D—C3D	118.8 (2)	C18H—C19H—H19Z	109.5
C5D—C4D—H4DA	120.6	H19Y—C19H—H19Z	109.5
C3D—C4D—H4DA	120.6	C18H—C19H—H191	109.5
C4D—C5D—C6D	121.0 (2)	H19Y—C19H—H191	109.5
C4D—C5D—H5DA	119.5	H19Z—C19H—H191	109.5
C6D—C5D—H5DA	119.5	C18H—C20H—H20Y	109.5
C1D—C6D—C5D	118.8 (2)	C18H—C20H—H20Z	109.5
C1D—C6D—C7D	122.42 (19)	H20Y—C20H—H20Z	109.5
C5D—C6D—C7D	118.81 (19)	C18H—C20H—H21V	109.5
N4D—C7D—C6D	120.3 (2)	H20Y—C20H—H21V	109.5
N4D—C7D—H7DA	119.9	H20Z—C20H—H21V	109.5
C6D—C7D—H7DA	119.9	C10H—C21H—H21W	109.5
N1D—C8D—N3D	103.06 (17)	C10H—C21H—H21X	109.5
N1D—C8D—S1D	127.89 (17)	H21W—C21H—H21X	109.5
N3D—C8D—S1D	128.94 (16)	C10H—C21H—H21Y	109.5
N2D—C9D—N3D	110.60 (18)	H21W—C21H—H21Y	109.5
N2D—C9D—C10D	126.23 (19)	H21X—C21H—H21Y	109.5
N3D—C9D—C10D	123.16 (19)	C8I—N1I—N2I	114.45 (18)
C9D—C10D—C11D	111.68 (17)	C8I—N1I—H1IB	122.8
C9D—C10D—C21D	110.64 (18)	N2I—N1I—H1IB	122.8
C11D—C10D—C21D	111.45 (16)	C9I—N2I—N1I	104.16 (17)
C9D—C10D—H10D	107.6	C8I—N3I—C9I	108.13 (17)
C11D—C10D—H10D	107.6	C8I—N3I—N4I	130.08 (16)
C21D—C10D—H10D	107.6	C9I—N3I—N4I	121.03 (16)
C16D—C11D—C12D	118.1 (2)	C7I—N4I—N3I	115.07 (17)
C16D—C11D—C10D	120.92 (18)	C2I—C1I—C6I	120.9 (2)
C12D—C11D—C10D	120.90 (19)	C2I—C1I—H1IA	119.5
C13D—C12D—C11D	120.6 (2)	C6I—C1I—H1IA	119.5
C13D—C12D—H12D	119.7	C1I—C2I—C3I	118.8 (2)
C11D—C12D—H12D	119.7	C1I—C2I—H2IA	120.6
C12D—C13D—C14D	121.4 (2)	C3I—C2I—H2IA	120.6
C12D—C13D—H13D	119.3	C4I—C3I—C2I	121.7 (2)
C14D—C13D—H13D	119.3	C4I—C3I—Cl1I	119.16 (17)
C15D—C14D—C13D	117.7 (2)	C2I—C3I—Cl1I	119.10 (18)
C15D—C14D—C17D	121.1 (2)	C5I—C4I—C3I	118.9 (2)
C13D—C14D—C17D	121.2 (2)	C5I—C4I—H4IA	120.5
C14D—C15D—C16D	121.2 (2)	C3I—C4I—H4IA	120.5
C14D—C15D—H15D	119.4	C4I—C5I—C6I	121.0 (2)
C16D—C15D—H15D	119.4	C4I—C5I—H5IA	119.5
C11D—C16D—C15D	120.9 (2)	C6I—C5I—H5IA	119.5
C11D—C16D—H16D	119.5	C1I—C6I—C5I	118.6 (2)
C15D—C16D—H16D	119.5	C1I—C6I—C7I	122.74 (19)
C14D—C17D—C18D	115.32 (19)	C5I—C6I—C7I	118.63 (19)
C14D—C17D—H17F	108.4	N4I—C7I—C6I	120.53 (19)
C18D—C17D—H17F	108.4	N4I—C7I—H7IA	119.7
C14D—C17D—H17G	108.4	C6I—C7I—H7IA	119.7
C18D—C17D—H17G	108.4	N1I—C8I—N3I	102.65 (17)
H17F—C17D—H17G	107.5	N1I—C8I—S1I	127.94 (17)
C20D—C18D—C17D	114.2 (2)	N3I—C8I—S1I	129.32 (16)
C20D—C18D—C19D	111.0 (2)	N2I—C9I—N3I	110.48 (18)
C17D—C18D—C19D	110.7 (2)	N2I—C9I—C10I	125.84 (19)
C20D—C18D—H18D	106.8	N3I—C9I—C10I	123.68 (19)
C17D—C18D—H18D	106.8	C9I—C10I—C11I	112.16 (16)
C19D—C18D—H18D	106.8	C9I—C10I—C21I	109.69 (18)
C18D—C19D—H19M	109.5	C11I—C10I—C21I	111.88 (17)
C18D—C19D—H19N	109.5	C9I—C10I—H10I	107.6
H19M—C19D—H19N	109.5	C11I—C10I—H10I	107.6
C18D—C19D—H19O	109.5	C21I—C10I—H10I	107.6
H19M—C19D—H19O	109.5	C16I—C11I—C12I	117.9 (2)
H19N—C19D—H19O	109.5	C16I—C11I—C10I	121.08 (18)
C18D—C20D—H20M	109.5	C12I—C11I—C10I	121.01 (19)
C18D—C20D—H20N	109.5	C13I—C12I—C11I	120.8 (2)
H20M—C20D—H20N	109.5	C13I—C12I—H12I	119.6
C18D—C20D—H20O	109.5	C11I—C12I—H12I	119.6
H20M—C20D—H20O	109.5	C12I—C13I—C14I	121.5 (2)
H20N—C20D—H20O	109.5	C12I—C13I—H13I	119.2
C10D—C21D—H21J	109.5	C14I—C13I—H13I	119.2
C10D—C21D—H21K	109.5	C13I—C14I—C15I	117.5 (2)
H21J—C21D—H21K	109.5	C13I—C14I—C17I	121.4 (2)
C10D—C21D—H21L	109.5	C15I—C14I—C17I	121.1 (2)
H21J—C21D—H21L	109.5	C16I—C15I—C14I	121.4 (2)
H21K—C21D—H21L	109.5	C16I—C15I—H15I	119.3
C8E—N1E—N2E	114.19 (17)	C14I—C15I—H15I	119.3
C8E—N1E—H1EB	122.9	C15I—C16I—C11I	120.9 (2)
N2E—N1E—H1EB	122.9	C15I—C16I—H16I	119.6
C9E—N2E—N1E	104.14 (17)	C11I—C16I—H16I	119.6
C8E—N3E—C9E	108.02 (17)	C14I—C17I—C18I	113.82 (18)
C8E—N3E—N4E	132.23 (16)	C14I—C17I—H17T	108.8
C9E—N3E—N4E	119.29 (16)	C18I—C17I—H17T	108.8
C7E—N4E—N3E	117.06 (17)	C14I—C17I—H17U	108.8
C2E—C1E—C6E	120.8 (2)	C18I—C17I—H17U	108.8
C2E—C1E—H1EA	119.6	H17T—C17I—H17U	107.7
C6E—C1E—H1EA	119.6	C20I—C18I—C19I	110.63 (19)
C1E—C2E—C3E	118.6 (2)	C20I—C18I—C17I	111.09 (19)
C1E—C2E—H2EA	120.7	C19I—C18I—C17I	110.35 (18)
C3E—C2E—H2EA	120.7	C20I—C18I—H18I	108.2
C4E—C3E—C2E	121.8 (2)	C19I—C18I—H18I	108.2
C4E—C3E—Cl1E	118.83 (19)	C17I—C18I—H18I	108.2
C2E—C3E—Cl1E	119.4 (2)	C18I—C19I—H192	109.5
C3E—C4E—C5E	119.2 (2)	C18I—C19I—H193	109.5
C3E—C4E—H4EA	120.4	H192—C19I—H193	109.5
C5E—C4E—H4EA	120.4	C18I—C19I—H194	109.5
C4E—C5E—C6E	120.6 (2)	H192—C19I—H194	109.5
C4E—C5E—H5EA	119.7	H193—C19I—H194	109.5
C6E—C5E—H5EA	119.7	C18I—C20I—H21Z	109.5
C1E—C6E—C5E	119.1 (2)	C18I—C20I—H22A	109.5
C1E—C6E—C7E	122.86 (19)	H21Z—C20I—H22A	109.5
C5E—C6E—C7E	118.05 (19)	C18I—C20I—H22B	109.5
N4E—C7E—C6E	119.29 (19)	H21Z—C20I—H22B	109.5
N4E—C7E—H7EA	120.4	H22A—C20I—H22B	109.5
C6E—C7E—H7EA	120.4	C10I—C21I—H22C	109.5
N1E—C8E—N3E	102.67 (17)	C10I—C21I—H22D	109.5
N1E—C8E—S1E	126.68 (16)	H22C—C21I—H22D	109.5
N3E—C8E—S1E	130.60 (16)	C10I—C21I—H22E	109.5
N2E—C9E—N3E	110.93 (18)	H22C—C21I—H22E	109.5
N2E—C9E—C10E	125.83 (19)	H22D—C21I—H22E	109.5
N3E—C9E—C10E	123.17 (19)		
			
C8A—N1A—N2A—C9A	−1.0 (2)	N4E—N3E—C8E—S1E	3.5 (3)
C8A—N3A—N4A—C7A	−38.0 (3)	N1E—N2E—C9E—N3E	−0.8 (2)
C9A—N3A—N4A—C7A	153.48 (18)	N1E—N2E—C9E—C10E	−177.80 (19)
C6A—C1A—C2A—C3A	0.0 (3)	C8E—N3E—C9E—N2E	1.9 (2)
C1A—C2A—C3A—C4A	0.6 (3)	N4E—N3E—C9E—N2E	175.02 (16)
C1A—C2A—C3A—Cl1A	−178.34 (16)	C8E—N3E—C9E—C10E	179.00 (18)
C2A—C3A—C4A—C5A	−0.8 (3)	N4E—N3E—C9E—C10E	−7.9 (3)
Cl1A—C3A—C4A—C5A	178.16 (15)	N2E—C9E—C10E—C11E	98.4 (2)
C3A—C4A—C5A—C6A	0.3 (3)	N3E—C9E—C10E—C11E	−78.3 (2)
C4A—C5A—C6A—C1A	0.2 (3)	N2E—C9E—C10E—C21E	−25.5 (3)
C4A—C5A—C6A—C7A	−178.57 (18)	N3E—C9E—C10E—C21E	157.83 (18)
C2A—C1A—C6A—C5A	−0.4 (3)	C9E—C10E—C11E—C16E	114.4 (2)
C2A—C1A—C6A—C7A	178.34 (19)	C21E—C10E—C11E—C16E	−123.0 (2)
N3A—N4A—C7A—C6A	179.79 (16)	C9E—C10E—C11E—C12E	−66.1 (2)
C5A—C6A—C7A—N4A	−170.67 (19)	C21E—C10E—C11E—C12E	56.5 (2)
C1A—C6A—C7A—N4A	10.6 (3)	C16E—C11E—C12E—C13E	0.9 (3)
N2A—N1A—C8A—N3A	2.5 (2)	C10E—C11E—C12E—C13E	−178.59 (18)
N2A—N1A—C8A—S1A	−173.89 (15)	C11E—C12E—C13E—C14E	−1.0 (3)
C9A—N3A—C8A—N1A	−2.9 (2)	C12E—C13E—C14E—C15E	0.6 (3)
N4A—N3A—C8A—N1A	−172.66 (18)	C12E—C13E—C14E—C17E	−179.68 (19)
C9A—N3A—C8A—S1A	173.38 (16)	C13E—C14E—C15E—C16E	−0.1 (3)
N4A—N3A—C8A—S1A	3.7 (3)	C17E—C14E—C15E—C16E	−179.88 (19)
N1A—N2A—C9A—N3A	−1.0 (2)	C14E—C15E—C16E—C11E	0.1 (3)
N1A—N2A—C9A—C10A	177.51 (19)	C12E—C11E—C16E—C15E	−0.5 (3)
C8A—N3A—C9A—N2A	2.6 (2)	C10E—C11E—C16E—C15E	179.01 (18)
N4A—N3A—C9A—N2A	173.44 (17)	C13E—C14E—C17E—C18E	95.5 (2)
C8A—N3A—C9A—C10A	−175.99 (18)	C15E—C14E—C17E—C18E	−84.7 (3)
N4A—N3A—C9A—C10A	−5.2 (3)	C14E—C17E—C18E—C20E	−63.8 (2)
N2A—C9A—C10A—C11A	102.4 (2)	C14E—C17E—C18E—C19E	173.6 (2)
N3A—C9A—C10A—C11A	−79.2 (2)	C8F—N1F—N2F—C9F	−1.1 (2)
N2A—C9A—C10A—C21A	−22.0 (3)	C8F—N3F—N4F—C7F	−27.3 (3)
N3A—C9A—C10A—C21A	156.34 (19)	C9F—N3F—N4F—C7F	160.66 (18)
C9A—C10A—C11A—C16A	119.5 (2)	C6F—C1F—C2F—C3F	0.4 (3)
C21A—C10A—C11A—C16A	−116.4 (2)	C1F—C2F—C3F—C4F	0.1 (3)
C9A—C10A—C11A—C12A	−63.0 (2)	C1F—C2F—C3F—Cl1F	179.98 (16)
C21A—C10A—C11A—C12A	61.2 (2)	C2F—C3F—C4F—C5F	−0.8 (3)
C16A—C11A—C12A—C13A	1.4 (3)	Cl1F—C3F—C4F—C5F	179.36 (16)
C10A—C11A—C12A—C13A	−176.23 (18)	C3F—C4F—C5F—C6F	0.9 (3)
C11A—C12A—C13A—C14A	−0.2 (3)	C4F—C5F—C6F—C1F	−0.4 (3)
C12A—C13A—C14A—C15A	−0.7 (3)	C4F—C5F—C6F—C7F	−178.90 (19)
C12A—C13A—C14A—C17A	177.0 (2)	C2F—C1F—C6F—C5F	−0.3 (3)
C13A—C14A—C15A—C16A	0.2 (3)	C2F—C1F—C6F—C7F	178.16 (19)
C17A—C14A—C15A—C16A	−177.4 (2)	N3F—N4F—C7F—C6F	−178.16 (16)
C14A—C15A—C16A—C11A	1.1 (3)	C5F—C6F—C7F—N4F	−167.62 (19)
C12A—C11A—C16A—C15A	−1.9 (3)	C1F—C6F—C7F—N4F	13.9 (3)
C10A—C11A—C16A—C15A	175.78 (18)	N2F—N1F—C8F—N3F	2.5 (2)
C13A—C14A—C17A—C18A	−72.5 (3)	N2F—N1F—C8F—S1F	−175.03 (15)
C15A—C14A—C17A—C18A	105.1 (3)	C9F—N3F—C8F—N1F	−2.8 (2)
C14A—C17A—C18A—C20A	−55.5 (3)	N4F—N3F—C8F—N1F	−175.50 (18)
C14A—C17A—C18A—C19A	178.3 (3)	C9F—N3F—C8F—S1F	174.51 (16)
C8B—N1B—N2B—C9B	1.1 (2)	N4F—N3F—C8F—S1F	1.9 (3)
C8B—N3B—N4B—C7B	26.7 (3)	N1F—N2F—C9F—N3F	−0.9 (2)
C9B—N3B—N4B—C7B	−160.85 (18)	N1F—N2F—C9F—C10F	178.77 (19)
C6B—C1B—C2B—C3B	−1.1 (3)	C8F—N3F—C9F—N2F	2.4 (2)
C1B—C2B—C3B—C4B	1.4 (3)	N4F—N3F—C9F—N2F	176.25 (16)
C1B—C2B—C3B—Cl1B	−178.41 (16)	C8F—N3F—C9F—C10F	−177.23 (18)
C2B—C3B—C4B—C5B	−0.1 (3)	N4F—N3F—C9F—C10F	−3.4 (3)
Cl1B—C3B—C4B—C5B	179.68 (16)	N2F—C9F—C10F—C11F	97.1 (2)
C3B—C4B—C5B—C6B	−1.5 (3)	N3F—C9F—C10F—C11F	−83.3 (2)
C2B—C1B—C6B—C5B	−0.5 (3)	N2F—C9F—C10F—C21F	−26.7 (3)
C2B—C1B—C6B—C7B	−178.14 (19)	N3F—C9F—C10F—C21F	152.90 (19)
C4B—C5B—C6B—C1B	1.8 (3)	C9F—C10F—C11F—C16F	118.0 (2)
C4B—C5B—C6B—C7B	179.50 (19)	C21F—C10F—C11F—C16F	−119.3 (2)
N3B—N4B—C7B—C6B	177.14 (16)	C9F—C10F—C11F—C12F	−64.9 (2)
C1B—C6B—C7B—N4B	−12.5 (3)	C21F—C10F—C11F—C12F	57.8 (2)
C5B—C6B—C7B—N4B	169.91 (18)	C16F—C11F—C12F—C13F	0.8 (3)
N2B—N1B—C8B—N3B	−2.4 (2)	C10F—C11F—C12F—C13F	−176.46 (18)
N2B—N1B—C8B—S1B	175.16 (15)	C11F—C12F—C13F—C14F	−0.5 (3)
C9B—N3B—C8B—N1B	2.8 (2)	C12F—C13F—C14F—C15F	−0.1 (3)
N4B—N3B—C8B—N1B	175.81 (18)	C12F—C13F—C14F—C17F	179.92 (19)
C9B—N3B—C8B—S1B	−174.68 (16)	C13F—C14F—C15F—C16F	0.3 (3)
N4B—N3B—C8B—S1B	−1.6 (3)	C17F—C14F—C15F—C16F	−179.69 (19)
N1B—N2B—C9B—N3B	0.8 (2)	C14F—C15F—C16F—C11F	0.0 (3)
N1B—N2B—C9B—C10B	−179.74 (19)	C12F—C11F—C16F—C15F	−0.6 (3)
C8B—N3B—C9B—N2B	−2.4 (2)	C10F—C11F—C16F—C15F	176.64 (18)
N4B—N3B—C9B—N2B	−176.51 (16)	C13F—C14F—C17F—C18F	97.2 (2)
C8B—N3B—C9B—C10B	178.19 (18)	C15F—C14F—C17F—C18F	−82.8 (3)
N4B—N3B—C9B—C10B	4.0 (3)	C14F—C17F—C18F—C20F	−63.3 (3)
N2B—C9B—C10B—C11B	−105.3 (2)	C14F—C17F—C18F—C19F	173.6 (2)
N3B—C9B—C10B—C11B	74.0 (2)	C8G—N1G—N2G—C9G	0.4 (2)
N2B—C9B—C10B—C21B	18.4 (3)	C8G—N3G—N4G—C7G	40.0 (3)
N3B—C9B—C10B—C21B	−162.29 (18)	C9G—N3G—N4G—C7G	−153.14 (18)
C9B—C10B—C11B—C16B	−125.0 (2)	C6G—C1G—C2G—C3G	1.2 (3)
C21B—C10B—C11B—C16B	112.1 (2)	C1G—C2G—C3G—C4G	−1.5 (3)
C9B—C10B—C11B—C12B	57.0 (3)	C1G—C2G—C3G—Cl1G	178.69 (16)
C21B—C10B—C11B—C12B	−65.9 (2)	C2G—C3G—C4G—C5G	0.9 (3)
C16B—C11B—C12B—C13B	0.2 (3)	Cl1G—C3G—C4G—C5G	−179.34 (16)
C10B—C11B—C12B—C13B	178.24 (18)	C3G—C4G—C5G—C6G	0.1 (3)
C11B—C12B—C13B—C14B	−0.1 (3)	C4G—C5G—C6G—C1G	−0.4 (3)
C12B—C13B—C14B—C15B	−0.1 (3)	C4G—C5G—C6G—C7G	179.42 (19)
C12B—C13B—C14B—C17B	−177.75 (19)	C2G—C1G—C6G—C5G	−0.2 (3)
C13B—C14B—C15B—C16B	0.1 (3)	C2G—C1G—C6G—C7G	179.93 (19)
C17B—C14B—C15B—C16B	177.7 (2)	N3G—N4G—C7G—C6G	−177.98 (16)
C12B—C11B—C16B—C15B	−0.2 (3)	C5G—C6G—C7G—N4G	175.69 (18)
C10B—C11B—C16B—C15B	−178.22 (18)	C1G—C6G—C7G—N4G	−4.5 (3)
C14B—C15B—C16B—C11B	0.0 (3)	N2G—N1G—C8G—N3G	−1.7 (2)
C13B—C14B—C17B—C18B	68.9 (3)	N2G—N1G—C8G—S1G	175.02 (15)
C15B—C14B—C17B—C18B	−108.6 (3)	C9G—N3G—C8G—N1G	2.2 (2)
C13B—C14B—C17B—C18X	108.6 (5)	N4G—N3G—C8G—N1G	170.47 (17)
C15B—C14B—C17B—C18X	−69.0 (6)	C9G—N3G—C8G—S1G	−174.39 (16)
C14B—C17B—C18B—C19B	45.6 (4)	N4G—N3G—C8G—S1G	−6.1 (3)
C18X—C17B—C18B—C19B	−58.0 (6)	N1G—N2G—C9G—N3G	1.1 (2)
C14B—C17B—C18B—C20B	175.5 (3)	N1G—N2G—C9G—C10G	179.70 (18)
C18X—C17B—C18B—C20B	72.0 (6)	C8G—N3G—C9G—N2G	−2.1 (2)
C14B—C17B—C18X—C19X	−23.2 (16)	N4G—N3G—C9G—N2G	−171.58 (16)
C18B—C17B—C18X—C19X	70.6 (14)	C8G—N3G—C9G—C10G	179.18 (18)
C14B—C17B—C18X—C20X	−172.3 (6)	N4G—N3G—C9G—C10G	9.7 (3)
C18B—C17B—C18X—C20X	−78.4 (8)	N2G—C9G—C10G—C11G	−102.0 (2)
C8C—N1C—N2C—C9C	−0.9 (2)	N3G—C9G—C10G—C11G	76.5 (2)
C8C—N3C—N4C—C7C	−26.4 (3)	N2G—C9G—C10G—C21G	22.2 (3)
C9C—N3C—N4C—C7C	161.12 (18)	N3G—C9G—C10G—C21G	−159.32 (18)
C6C—C1C—C2C—C3C	0.0 (3)	C9G—C10G—C11G—C16G	−119.3 (2)
C1C—C2C—C3C—C4C	0.1 (3)	C21G—C10G—C11G—C16G	117.4 (2)
C1C—C2C—C3C—Cl1C	179.25 (16)	C9G—C10G—C11G—C12G	61.4 (2)
C2C—C3C—C4C—C5C	−0.7 (3)	C21G—C10G—C11G—C12G	−61.9 (2)
Cl1C—C3C—C4C—C5C	−179.90 (16)	C16G—C11G—C12G—C13G	0.5 (3)
C3C—C4C—C5C—C6C	1.3 (3)	C10G—C11G—C12G—C13G	179.80 (18)
C4C—C5C—C6C—C1C	−1.1 (3)	C11G—C12G—C13G—C14G	0.0 (3)
C4C—C5C—C6C—C7C	−179.52 (18)	C12G—C13G—C14G—C15G	−0.6 (3)
C2C—C1C—C6C—C5C	0.4 (3)	C12G—C13G—C14G—C17G	178.9 (2)
C2C—C1C—C6C—C7C	178.83 (19)	C13G—C14G—C15G—C16G	0.8 (3)
N3C—N4C—C7C—C6C	−177.29 (16)	C17G—C14G—C15G—C16G	−178.7 (2)
C5C—C6C—C7C—N4C	−167.63 (18)	C14G—C15G—C16G—C11G	−0.4 (3)
C1C—C6C—C7C—N4C	14.0 (3)	C12G—C11G—C16G—C15G	−0.2 (3)
N2C—N1C—C8C—N3C	2.4 (2)	C10G—C11G—C16G—C15G	−179.59 (18)
N2C—N1C—C8C—S1C	−175.25 (15)	C15G—C14G—C17G—C18G	−75.6 (3)
C9C—N3C—C8C—N1C	−2.9 (2)	C13G—C14G—C17G—C18G	104.9 (3)
N4C—N3C—C8C—N1C	−175.97 (19)	C14G—C17G—C18G—C20G	−41.9 (5)
C9C—N3C—C8C—S1C	174.67 (16)	C14G—C17G—C18G—C19G	−177.7 (3)
N4C—N3C—C8C—S1C	1.6 (3)	C8H—N1H—N2H—C9H	−1.1 (2)
N1C—N2C—C9C—N3C	−1.0 (2)	C8H—N3H—N4H—C7H	−33.6 (3)
N1C—N2C—C9C—C10C	−179.25 (19)	C9H—N3H—N4H—C7H	156.05 (18)
C8C—N3C—C9C—N2C	2.6 (2)	C6H—C1H—C2H—C3H	0.0 (3)
N4C—N3C—C9C—N2C	176.81 (16)	C1H—C2H—C3H—C4H	1.4 (3)
C8C—N3C—C9C—C10C	−179.15 (18)	C1H—C2H—C3H—Cl1H	−177.98 (16)
N4C—N3C—C9C—C10C	−4.9 (3)	C2H—C3H—C4H—C5H	−1.5 (3)
N2C—C9C—C10C—C11C	106.1 (2)	Cl1H—C3H—C4H—C5H	177.87 (16)
N3C—C9C—C10C—C11C	−71.9 (2)	C3H—C4H—C5H—C6H	0.2 (3)
N2C—C9C—C10C—C21C	−17.3 (3)	C2H—C1H—C6H—C5H	−1.2 (3)
N3C—C9C—C10C—C21C	164.63 (18)	C2H—C1H—C6H—C7H	177.29 (19)
C9C—C10C—C11C—C16C	130.7 (2)	C4H—C5H—C6H—C1H	1.0 (3)
C21C—C10C—C11C—C16C	−106.5 (2)	C4H—C5H—C6H—C7H	−177.50 (18)
C9C—C10C—C11C—C12C	−51.4 (3)	N3H—N4H—C7H—C6H	−179.00 (16)
C21C—C10C—C11C—C12C	71.4 (2)	C1H—C6H—C7H—N4H	7.3 (3)
C16C—C11C—C12C—C13C	−0.1 (3)	C5H—C6H—C7H—N4H	−174.28 (19)
C10C—C11C—C12C—C13C	−178.07 (18)	N2H—N1H—C8H—N3H	2.6 (2)
C11C—C12C—C13C—C14C	0.1 (3)	N2H—N1H—C8H—S1H	−174.78 (15)
C12C—C13C—C14C—C15C	−0.1 (3)	C9H—N3H—C8H—N1H	−2.9 (2)
C12C—C13C—C14C—C17C	177.3 (2)	N4H—N3H—C8H—N1H	−174.13 (18)
C13C—C14C—C15C—C16C	0.0 (3)	C9H—N3H—C8H—S1H	174.33 (16)
C17C—C14C—C15C—C16C	−177.4 (2)	N4H—N3H—C8H—S1H	3.1 (3)
C14C—C15C—C16C—C11C	0.0 (3)	N1H—N2H—C9H—N3H	−0.9 (2)
C12C—C11C—C16C—C15C	0.1 (3)	N1H—N2H—C9H—C10H	179.89 (19)
C10C—C11C—C16C—C15C	177.99 (19)	C8H—N3H—C9H—N2H	2.5 (2)
C13C—C14C—C17C—C18C	−63.8 (3)	N4H—N3H—C9H—N2H	174.82 (16)
C15C—C14C—C17C—C18C	113.5 (3)	C8H—N3H—C9H—C10H	−178.25 (18)
C14C—C17C—C18C—C19C	−50.4 (3)	N4H—N3H—C9H—C10H	−5.9 (3)
C14C—C17C—C18C—C20C	−175.1 (2)	N2H—C9H—C10H—C11H	98.5 (2)
C8D—N1D—N2D—C9D	0.6 (2)	N3H—C9H—C10H—C11H	−80.7 (2)
C8D—N3D—N4D—C7D	38.7 (3)	N2H—C9H—C10H—C21H	−26.9 (3)
C9D—N3D—N4D—C7D	−153.84 (18)	N3H—C9H—C10H—C21H	153.90 (19)
C6D—C1D—C2D—C3D	1.1 (3)	C9H—C10H—C11H—C12H	−72.8 (2)
C1D—C2D—C3D—C4D	−1.1 (3)	C21H—C10H—C11H—C12H	51.1 (3)
C1D—C2D—C3D—Cl1D	179.03 (16)	C9H—C10H—C11H—C16H	108.7 (2)
C2D—C3D—C4D—C5D	0.7 (3)	C21H—C10H—C11H—C16H	−127.5 (2)
Cl1D—C3D—C4D—C5D	−179.51 (16)	C16H—C11H—C12H—C13H	0.3 (3)
C3D—C4D—C5D—C6D	−0.1 (3)	C10H—C11H—C12H—C13H	−178.33 (18)
C2D—C1D—C6D—C5D	−0.6 (3)	C11H—C12H—C13H—C14H	0.3 (3)
C2D—C1D—C6D—C7D	179.71 (19)	C12H—C13H—C14H—C15H	−0.5 (3)
C4D—C5D—C6D—C1D	0.1 (3)	C12H—C13H—C14H—C17H	−179.49 (19)
C4D—C5D—C6D—C7D	179.81 (19)	C13H—C14H—C15H—C16H	0.1 (3)
N3D—N4D—C7D—C6D	−179.68 (16)	C17H—C14H—C15H—C16H	179.10 (19)
C1D—C6D—C7D—N4D	−6.8 (3)	C14H—C15H—C16H—C11H	0.5 (3)
C5D—C6D—C7D—N4D	173.47 (19)	C12H—C11H—C16H—C15H	−0.7 (3)
N2D—N1D—C8D—N3D	−2.2 (2)	C10H—C11H—C16H—C15H	177.94 (18)
N2D—N1D—C8D—S1D	174.31 (15)	C13H—C14H—C17H—C18H	101.3 (2)
C9D—N3D—C8D—N1D	2.9 (2)	C15H—C14H—C17H—C18H	−77.6 (3)
N4D—N3D—C8D—N1D	171.54 (18)	C14H—C17H—C18H—C20H	−60.8 (2)
C9D—N3D—C8D—S1D	−173.61 (16)	C14H—C17H—C18H—C19H	176.25 (19)
N4D—N3D—C8D—S1D	−4.9 (3)	C8I—N1I—N2I—C9I	−1.0 (2)
N1D—N2D—C9D—N3D	1.3 (2)	C8I—N3I—N4I—C7I	−39.2 (3)
N1D—N2D—C9D—C10D	−178.53 (19)	C9I—N3I—N4I—C7I	151.99 (18)
C8D—N3D—C9D—N2D	−2.7 (2)	C6I—C1I—C2I—C3I	−0.5 (3)
N4D—N3D—C9D—N2D	−172.69 (16)	C1I—C2I—C3I—C4I	1.4 (3)
C8D—N3D—C9D—C10D	177.12 (18)	C1I—C2I—C3I—Cl1I	−178.51 (16)
N4D—N3D—C9D—C10D	7.2 (3)	C2I—C3I—C4I—C5I	−1.3 (3)
N2D—C9D—C10D—C11D	−107.3 (2)	Cl1I—C3I—C4I—C5I	178.67 (16)
N3D—C9D—C10D—C11D	72.9 (2)	C3I—C4I—C5I—C6I	0.2 (3)
N2D—C9D—C10D—C21D	17.5 (3)	C2I—C1I—C6I—C5I	−0.5 (3)
N3D—C9D—C10D—C21D	−162.32 (18)	C2I—C1I—C6I—C7I	178.65 (19)
C9D—C10D—C11D—C16D	−120.5 (2)	C4I—C5I—C6I—C1I	0.7 (3)
C21D—C10D—C11D—C16D	115.2 (2)	C4I—C5I—C6I—C7I	−178.52 (19)
C9D—C10D—C11D—C12D	62.1 (2)	N3I—N4I—C7I—C6I	179.33 (16)
C21D—C10D—C11D—C12D	−62.3 (2)	C1I—C6I—C7I—N4I	6.3 (3)
C16D—C11D—C12D—C13D	−0.4 (3)	C5I—C6I—C7I—N4I	−174.55 (19)
C10D—C11D—C12D—C13D	177.09 (18)	N2I—N1I—C8I—N3I	3.0 (2)
C11D—C12D—C13D—C14D	−0.8 (3)	N2I—N1I—C8I—S1I	−173.70 (16)
C12D—C13D—C14D—C15D	1.0 (3)	C9I—N3I—C8I—N1I	−3.7 (2)
C12D—C13D—C14D—C17D	−178.55 (19)	N4I—N3I—C8I—N1I	−173.59 (18)
C13D—C14D—C15D—C16D	−0.1 (3)	C9I—N3I—C8I—S1I	172.94 (16)
C17D—C14D—C15D—C16D	179.48 (19)	N4I—N3I—C8I—S1I	3.0 (3)
C12D—C11D—C16D—C15D	1.3 (3)	N1I—N2I—C9I—N3I	−1.5 (2)
C10D—C11D—C16D—C15D	−176.17 (18)	N1I—N2I—C9I—C10I	177.74 (19)
C14D—C15D—C16D—C11D	−1.1 (3)	C8I—N3I—C9I—N2I	3.4 (2)
C15D—C14D—C17D—C18D	−117.8 (2)	N4I—N3I—C9I—N2I	174.36 (17)
C13D—C14D—C17D—C18D	61.7 (3)	C8I—N3I—C9I—C10I	−175.84 (18)
C14D—C17D—C18D—C20D	54.2 (3)	N4I—N3I—C9I—C10I	−4.9 (3)
C14D—C17D—C18D—C19D	−179.6 (2)	N2I—C9I—C10I—C11I	97.1 (2)
C8E—N1E—N2E—C9E	−0.6 (2)	N3I—C9I—C10I—C11I	−83.8 (2)
C8E—N3E—N4E—C7E	−29.4 (3)	N2I—C9I—C10I—C21I	−27.9 (3)
C9E—N3E—N4E—C7E	159.46 (18)	N3I—C9I—C10I—C21I	151.21 (19)
C6E—C1E—C2E—C3E	0.4 (3)	C9I—C10I—C11I—C16I	107.3 (2)
C1E—C2E—C3E—C4E	0.3 (3)	C21I—C10I—C11I—C16I	−129.0 (2)
C1E—C2E—C3E—Cl1E	−178.77 (16)	C9I—C10I—C11I—C12I	−75.5 (2)
C2E—C3E—C4E—C5E	−0.7 (3)	C21I—C10I—C11I—C12I	48.3 (3)
Cl1E—C3E—C4E—C5E	178.35 (17)	C16I—C11I—C12I—C13I	1.0 (3)
C3E—C4E—C5E—C6E	0.4 (3)	C10I—C11I—C12I—C13I	−176.34 (19)
C2E—C1E—C6E—C5E	−0.7 (3)	C11I—C12I—C13I—C14I	0.1 (3)
C2E—C1E—C6E—C7E	177.17 (19)	C12I—C13I—C14I—C15I	−0.9 (3)
C4E—C5E—C6E—C1E	0.3 (3)	C12I—C13I—C14I—C17I	−179.87 (19)
C4E—C5E—C6E—C7E	−177.70 (19)	C13I—C14I—C15I—C16I	0.5 (3)
N3E—N4E—C7E—C6E	−178.41 (16)	C17I—C14I—C15I—C16I	179.47 (19)
C1E—C6E—C7E—N4E	10.7 (3)	C14I—C15I—C16I—C11I	0.7 (3)
C5E—C6E—C7E—N4E	−171.40 (19)	C12I—C11I—C16I—C15I	−1.4 (3)
N2E—N1E—C8E—N3E	1.7 (2)	C10I—C11I—C16I—C15I	175.94 (18)
N2E—N1E—C8E—S1E	−175.93 (15)	C13I—C14I—C17I—C18I	105.0 (2)
C9E—N3E—C8E—N1E	−2.1 (2)	C15I—C14I—C17I—C18I	−73.9 (3)
N4E—N3E—C8E—N1E	−173.99 (18)	C14I—C17I—C18I—C20I	−59.6 (3)
C9E—N3E—C8E—S1E	175.43 (17)	C14I—C17I—C18I—C19I	177.32 (19)

### Hydrogen-bond geometry (Å, °)

**Table:**

*D*—H···*A*	*D*—H	H···*A*	*D*···*A*	*D*—H···*A*
N1A—H1AB···S1F^i^	0.86	2.52	3.373 (2)	170
N1B—H1BB···S1C^ii^	0.86	2.47	3.3072 (19)	163
N1C—H1CB···S1B^iii^	0.86	2.45	3.285 (2)	165
N1D—H1DB···S1E^iv^	0.86	2.48	3.3289 (19)	171
N1E—H1EB···S1D^v^	0.86	2.41	3.253 (2)	168
N1F—H1FB···S1A^i^	0.86	2.41	3.256 (2)	167
N1G—H1GB···S1H^iv^	0.86	2.43	3.287 (2)	174
N1H—H1HB···S1G^v^	0.86	2.45	3.3047 (19)	170
N1I—H1IB···S1I^vi^	0.86	2.45	3.301 (2)	172
C10D—H10D···S1C^iv^	0.98	2.87	3.769 (2)	152
C10G—H10G···S1F^iv^	0.98	2.82	3.662 (2)	144
C7A—H7AA···S1A	0.93	2.58	3.174 (2)	122
C7B—H7BA···S1B	0.93	2.57	3.240 (2)	130
C7C—H7CA···S1C	0.93	2.58	3.254 (2)	130
C7D—H7DA···S1D	0.93	2.59	3.174 (2)	121
C7E—H7EA···S1E	0.93	2.56	3.223 (2)	129
C7F—H7FA···S1F	0.93	2.56	3.229 (2)	130
C7G—H7GA···S1G	0.93	2.61	3.178 (2)	120
C7H—H7HA···S1H	0.93	2.55	3.187 (2)	126
C7I—H7IA···S1I	0.93	2.61	3.179 (2)	120
C4D—H4DA···Cg1	0.93	2.72	3.588 (2)	156
C4E—H4EA···Cg2	0.93	2.84	3.566 (3)	136
C4G—H4GA···Cg3	0.93	2.86	3.701 (2)	152
C4H—H4HA···Cg4	0.93	2.51	3.338 (2)	148
C4A—H4AA···Cg5^vi^	0.93	2.72	3.606 (2)	159
C4C—H4CA···Cg6^vii^	0.93	2.96	3.670 (2)	134
C4F—H4FA···Cg7^vi^	0.93	2.78	3.524 (3)	137
C4I—H4IA···Cg8^viii^	0.93	2.88	3.719 (2)	151

Symmetry codes: (i) −*x*, −*y*+1, −*z*+1; (ii) *x*+1, *y*+1, *z*; (iii) *x*−1, *y*−1, *z*; (iv) *x*+1, *y*, *z*; (v) *x*−1, *y*, *z*; (vi) −*x*+1, −*y*+1, −*z*+1; (vii) *x*, *y*−1, *z*; (viii) −*x*+2, −*y*+1, −*z*+1.
